# Adverse pro-tumorigenic effects of IDO1 catalytic inhibitors mediated by the non-enzymatic function of IDO1 in tumor cells

**DOI:** 10.3389/fimmu.2025.1680896

**Published:** 2025-11-04

**Authors:** Sofia Rossini, Sara Ambrosino, Claudia Volpi, Chiara Suvieri, Maria Teresa Pallotta, Maria Laura Belladonna, Daniele Sorcini, Antonio Macchiarulo, Elisabeth Hennes, Slava Ziegler, Herbert Waldmann, Ciriana Orabona

**Affiliations:** ^1^ Department of Medicine and Surgery, University of Perugia, Perugia, Italy; ^2^ Department of Pharmaceutical Sciences, University of Perugia, Perugia, Italy; ^3^ Department of Chemical Biology, Max Planck Institute of Molecular Physiology, Dortmund, Germany

**Keywords:** apo-IDO1, non-enzymatic function of IDO1, IDO1 protein degradation, epacadostat, linrodostat, navoximod, human thyroid cancer cells

## Abstract

Indoleamine 2,3-dioxygenase 1 (IDO1) inhibitors have been developed with the aim of reinvigorating antitumor T-cell responses in the tumor microenvironment by blocking the conversion of the essential amino acid tryptophan into immunoregulatory kynurenines. The lack of efficacy demonstrated in the clinical trials prompts us to revise the “on-target” mechanism of these molecules. By studying the turnover of IDO1 protein in human tumor cells exposed to various IDO1 catalytic inhibitors, such as epacadostat, linrodostat, and navoximod, we show here that these molecules stabilize a non-enzymatic protein conformation of IDO1, independently of their mechanism of inhibition. In the thyroid carcinoma cell line FTC-133, the stabilized and non-enzymatic IDO1 protein promotes the proliferation and migration of the tumor, resulting in an adverse pro-tumorigenic effect. These results uncover an unexpected adverse effect of IDO1 inhibitors in the tumor microenvironment that overcomes the enzymatic inhibition of IDO1, and suggest protein degradation, rather than enzymatic inhibition, as a more effective approach to target IDO1 in the tumor microenvironment.

## Introduction

The catalytic inhibitors of the immunoregulatory enzyme indoleamine 2,3-dioxygenase 1 (IDO1) are investigational drugs for cancer that have been developed with the rationale of reinvigorating the immune responses against the tumor. Preclinical evidence suggested that the conversion of the essential amino acid tryptophan (Trp) to immunoregulatory kynurenines (Kyn), generated by the enzymatic activity of IDO1, represents a key mechanism of immune escape within the tumor microenvironment (TME) (1, 2). Indeed, the synergistic IDO1-mediated Trp depletion and Kyn enrichment within the TME increase T regulatory cell (Treg) and myeloid-derived suppressor cell (MDSC) infiltration, induce differentiation of monocytes into M2 macrophages, decrease dendritic cell (DC) antigen uptake, and impair cytotoxic T-cell function (3–6), with a resultant immunosuppressive microenvironment that prevents an effective antitumor response.

Numerous IDO1 inhibitors have been introduced into clinical trials as immunotherapeutic agents for cancer treatment to block the Trp–Kyn pathway. Most are small molecules that efficiently inhibit the catalytic activity of IDO1 by both competitive and non-competitive mechanisms and, more recently, by displacing the cofactor heme, which is essential for IDO1 enzymatic activity, with a resulting decrease in the Kyn/Trp ratio in the TME (7, 8). With the rationale to block this metabolic mechanism of immune evasion in the TME, IDO1 catalytic inhibitors reached clinical trials, and some of these molecules, such as epacadostat, linrodostat, and navoximod, entered phase III as monotherapy or in combination with different immunotherapeutic agents (9). Despite the solid immunotherapeutic rationale of IDO1 inhibitors, the reversal of tumoral immune resistance by IDO1 catalytic activity inhibition did not meet clinical efficacy, and several phase III trials were scaled back or discontinued (10).

Although several justifications were given for these disappointing results, to date, the reasons for the failure are still under debate (11). Interestingly, recent observations demonstrated a dual function for IDO1, conferring on this protein a moonlighting feature (12, 13). Indeed, the IDO1 protein has a non-enzymatic function that makes it an intracellular signaling molecule capable of triggering different cell-specific pathways. IDO1’s signaling function is facilitated by a protein conformation that exposes two highly conserved immunoreceptor tyrosine-based inhibitory motifs (ITIMs), allowing them to be phosphorylated and acting as docking sites for interaction with SH2-containing proteins responsible for the downstream signal transduction. Moreover, Hoffka et al. recently described the conformational changes of ITIM-phosphorylated IDO1, suggesting ITIM phosphorylation as a sort of switch to balance enzymatic versus non-enzymatic IDO1 (14). The IDO1 partners interacting with phosphorylated ITIM1 and ITIM2 were first characterized in DCs, where they control the turnover of the protein and consequently affect the DC phenotype (15). Among them, the suppressor of cytokine signaling 3 (SOCS3) drives the proteasomal degradation of IDO1 in response to inflammatory conditions, interacting with ITIM2 (16), and abrogates the IDO1-dependent tolerogenesis in DCs, forcing the expression of an immunogenic phenotype (17). By contrast, in an immunosuppressive TGF-β-driven microenvironment, the IDO1-mediated signal transduction in DCs involves upstream the interaction of ITIM1 (16) with Src homology 2 domain phosphatases (SHPs) that culminates downstream with *de novo* transcription of *Ido1* and *Tgfb1* genes, reinforcing the tolerogenic phenotype in DCs (18). Recently, the signaling pathways triggered by the IDO1 protein have also been explored in IDO1-expressing tumor cells. We demonstrated that the B16 melanoma expressing a loss-of-function mutant of IDO1 catalytic activity proliferated faster than the wild-type tumor and expressed a constitutive hyperactivated Ras–ERK signaling pathway (19), demonstrating that the non-enzymatic function of IDO1 was also relevant for tumor proliferation. Interestingly, the most clinically advanced catalytic inhibitor epacadostat stabilized a non-enzymatic conformation of IDO1 and fostered the proliferation of SKOV-3 cells, a human ovarian cancer cell line constitutively expressing IDO1 (20). Overall, our recent observations in tumor cells indicated a pro-tumorigenic adverse effect of IDO1 catalytic inhibition attributable to the non-enzymatic (i.e., signaling) function of IDO1 in tumor cells.

Here, we extended the pro-tumorigenic adverse effect of IDO1 catalytic inhibition to different human IDO1-expressing tumor cell lines, dissecting the signaling pathway mediated by IDO1 and proposing IDO1 protein degradation as an effective approach to neutralize the dual function of IDO1 in cancer.

## Materials and methods

### Cell lines, cell treatments, and reagents

The human ovarian adenocarcinoma cell line SKOV-3 was purchased from CLS Cell Lines Service GmbH, Eppelheim, GERMANY. The human follicular thyroid carcinoma cell line FTC-133 and the human papillary thyroid carcinoma cell line B-CPAP were kindly provided by Professor Puxeddu (University of Perugia, Perugia, Italy), whereas the human B-cell lymphoma cell line RC-K8 was kindly provided by Professor Sportoletti (University of Perugia, Perugia, Italy). SKOV-3, B-CPAP, and RC-K8 cell lines were grown in RPMI-1640 medium (Thermo Fisher Scientific, Waltham, MA,USA), whereas FTC-133 was grown in DMEM/F12 medium (Euroclone S.p.A., Milano, ITALY), both supplemented with 10% fetal calf serum (FCS). All the cell lines were maintained in a humidified atmosphere at 37°C in 5% CO_2_ and were routinely screened to confirm the absence of mycoplasma contamination. For cellular assays, unless otherwise specified, SKOV-3, FTC-133, and B-CPAP cells were seeded at a final concentration of 0.15 × 10^6^ cells/mL in a 12-well plate, whereas RC-K8 cells were cultured at 0.5 × 10^6^ cells/mL in a 24-well plate, and then exposed to IDO1 catalytic inhibitors or iDeg-1 for the indicated times. For the cycloheximide chase assay, SKOV-3 and FTC-133 cells were pretreated with the protein synthesis inhibitor for 1 h prior to the addition of IDO1 catalytic inhibitors or vehicle and then incubated up to 8 or 16 h, depending on the experiment. Epacadostat (CAS No. 1204669-58-8) and navoximod (CAS No. 1402837-78-8) were purchased from Selleck Chemicals (Houston, TX, USA), whereas linrodostat (CAS No. 1923833-60-6) was obtained from Chemgood, Henrico, VA, USA. All the IDO1 catalytic inhibitors were used at the final concentration of 1 µM, a concentration previously shown to be non-cytotoxic, sufficient to inhibit IDO1 catalytic activity, and able to promote IDO1 protein stabilization (20). Although higher than their nanomolar IC_50_ values, this concentration was chosen to ensure both effects while minimizing potential AhR-mediated transcriptional bias. Cycloheximide was obtained from Sigma-Aldrich, Merck, Darmstadt, GERMANY and used at a final concentration of 50 µg/mL. iDeg-1 was kindly provided by Professor Waldmann (Max Planck Institute of Molecular Physiology, Dortmund, Germany) and used at the final concentration of 10 µM in line with its initial characterization in tumor cell lines (21). DMSO, used as a vehicle control, was purchased from Sigma-Aldrich, Merck, Darmstadt, GERMANY. For the cell-free assay, recombinant Src protein (SRC, active, GST-tagged human) and recombinant human IDO1 protein (residues 1–403) were obtained from Sigma-Aldrich, Merck,Darmstadt, GERMANY and Giotto Biotech S.r.l., Firenze, ITALY, respectively. IDO1 knockdown experiments were performed by using Silencer Select human *IDO1* siRNA (ID s7425) and Silencer Select Negative Control No. 1 siRNA (Thermo Fischer Scientific, Waltham, MA, USA), according to the manufacturer’s instructions. Briefly, FTC-133 cells were seeded at a final concentration of 0.1 × 10^6^ cells/mL in 2 mL in a 6-well plate and cultured overnight. The day after, cells were transfected with 75 pmol of IDO1-specific or negative control (n.c.) siRNAs, by using Lipofectamine™ 3000 Transfection Reagent (Thermo Fischer Scientific, Waltham, MA,USA), and incubated for the indicated times. Specifically, transfected cells were incubated for 48 h prior to performing the scratch wound healing or the transwell migration assay, as described below.

### Immunoblot and kynurenine measurement

IDO1 protein expression was analyzed by means of a rabbit monoclonal anti-human IDO1 antibody (D5J4E™, Cell Signaling Technology, Danvers, MA, USA), whereas the SHP-2 protein level was detected using a mouse monoclonal anti -SH-PTP2 antibody (clone B-1, Santa Cruz Biotechnology, Dallas, TX, USA). The anti-phospho-tyrosine (p-Tyr-1000) MultiMab™ Rabbit monoclonal antibody mix (Cell Signaling Technology, Danvers, MA, USA) was used to detect the tyrosine-phosphorylation of IDO1, whereas a rabbit anti-phospho-Src family (Tyr416) antibody (Cell Signaling Technology, Danvers, MA, USA), followed by a rabbit monoclonal anti-Src antibody (36D10, Cell Signaling Technology, Danvers, MA, USA), was used to evaluate the p-Src/Src ratio. Moreover, a mouse monoclonal anti-β-tubulin antibody (clone AA2, Sigma-Aldrich, Merck, Darmstadt, GERMANY), a mouse monoclonal anti-actin antibody (clone AC-40, Sigma-Aldrich), and a rabbit monoclonal anti-GAPDH antibody (14C10, Cell Signaling Technology, Danvers, MA, USA) were used as normalizers, as appropriate. After incubation with the specific horseradish peroxidase-conjugated secondary antibodies (Sigma-Aldrich, Merck, Darmstadt, GERMANY) and the addition of a Clarity™ Western ECL (Enhanced ChemiLuminescence, Bio-Rad Laboratories, Hercules, CA, USA) substrate, signals were detected using the ChemiDoc™ MP Imaging System (Bio-Rad Laboratories, Hercules, CA, USA). Protein expression was quantified by densitometric analysis, using ImageLab Software (Bio-Rad Laboratories, Hercules, CA, USA), as previously described (20). The enzymatic activity of IDO1, which is the ability of the enzyme to convert Trp to Kyn, was evaluated in cell culture supernatants by HPLC, as previously described (22).

### Immunoprecipitation experiments

IDO1 tyrosine-phosphorylation and its interaction with Src or SHP-2 were assessed by immunoprecipitation experiments in FTC-133 cells. For each condition, 3 × 10^6^ cells were preconditioned with IDO1 catalytic inhibitors at 37°C and lysed in a lysis buffer (50 mM Tris–HCl, 150 mM NaCl, 1% Nonidet P-40, pH 7.5) containing protease and phosphatase inhibitor cocktails (Thermo Fischer Scientific, Waltham, MA, USA). After lysis, a representative aliquot per sample was collected as a whole cell lysate (WCL) control, whereas the remaining lysates were incubated overnight at 4°C under rotary agitation, in the presence of the specific antibody. Lysates from vehicle-treated cells, incubated without the specific antibody, were used as negative controls. The Protein G Sepharose™ 4 Fast Flow (Cytiva, Milano, ITALY) was preblocked overnight at 4°C with PBS containing 5% BSA (w/v) and then added to the lysates, including the negative control (i.e., only resin). After 1 h at 4°C, under rotary agitation, the immuno-complexes were isolated by centrifugation, eluted with Laemmli buffer containing 2-mercaptoethanol, and used for immunoblot analysis. Prior to performing quantitative analysis, aspecific signals detected in the negative control (n.c.) were subtracted from the specific ones.

### Src kinase activity

For monitoring IDO1 phosphorylation by Src kinase in the cell-free system, 300 ng of rhIDO1 were preconditioned with 10 µM of IDO1 catalytic inhibitors at 25°C for 30 min and then exposed to 5 ng of rhSrc for an additional 60 min. The reaction was performed in the presence of 100 µM ATP, in an assay buffer containing 100 mM Tris–HCl (pH 7.2), 125 mM MgCl_2_, 25 mM MnCl_2_, 250 µM Na_3_VO_2_, and 2 mM DTT. For measurement of Src kinase activity, 50 µM of a substrate synthetic peptide, corresponding to amino acids 6–20 of p34cdc2 (peptide sequence: KVEKIGEGTYGVVYK), was added, and ADP production was quantified using the ADP-Glo™ Kinase Assay Kit (Promega, Milano, ITALY), according to the manufacturer’s protocol. The resulting luminescence was measured with a plate-reading luminometer (Tecan, Spark^®, Mannedorf, SWISS^). For the determination of IDO1 and Src phosphorylation, the reaction was stopped by the addition of Laemmli buffer containing 2-mercaptoethanol and analyzed by SDS-PAGE.

### Real-time PCR analysis

For gene expression analysis, the human cancer cell lines (i.e., SKOV-3, FTC-133, B-CPAP, and RC-K8) were treated with IDO1 catalytic inhibitors for 24 h. Cells were lysed in TRIzol reagent (Thermo Fischer Scientific, Waltham, MA, USA) to isolate RNA, according to the manufacturer’s instructions, and 1 µg of RNA was reverse-transcribed using the QuantiTec Reverse Transcription Kit (Qiagen, Hilden, GERMANY). cDNAs were analyzed by real-time PCR using the QuantStudio 1 Real-Time PCR System (Applied Biosystems, Thermo Fisher Scientific, Waltham, MA, USA). Specific primers were used to determine the expression of the human *IDO1* gene (sense, 5′-TCACAGACCACAAGTCACAG-3′; anti-sense, 5′-GCAAGACCTTACGGACATCT-3′). Data were calculated as the ratio of the expression of the gene of interest to that of human β*-*actin (*ACTB*) (sense, 5′-CTCGTCGTCGACAACGGCT-3′; anti-sense, 5′-TCAGGGTGAGGATGCCTCTC-3′) by the relative quantification method (ΔΔCt). Values are presented as normalized transcript expression in samples relative to normalized transcript expression in control cells (1-fold).

### Proximity ligation assay

*In situ* proximity ligation assay (PLA) was performed using the NaveniFlex™ Cell Red Kit (Navinci Diagnostics, Uppsala, SWEDEN), according to the manufacturer’s instructions. Briefly, 0.2 × 10^6^ FTC-133 cells were seeded over a 24 mm cover glass in a 6-well plate and preconditioned with IDO1 catalytic inhibitors or vehicle. Then, cells were fixed with 4% paraformaldehyde (PFA) for 15 min at RT, permeabilized with 0.1% Triton X-100 in phosphate-buffered saline (PBS) for 10 min at RT, and blocked using Naveni Block reagent for 60 min at 37°C in a humidity chamber. Cells were then incubated with the specific pair of primary antibodies overnight at 4°C in the humidity chamber. Specifically, a rabbit monoclonal anti-human IDO1 antibody (D5J4E™, Cell Signaling Technology, Danvers, MA, USA), a mouse monoclonal anti-Src antibody (17AT28, Thermo Fisher Scientific, Waltham, MA, USA), and a mouse monoclonal anti-SH-PTP2 antibody (clone B-1, Santa Cruz Biotechnology, Dallas, TX, USA) were combined to detect IDO1/Src or IDO1/SHP-2 interactions. The day after, incubation with the specific oligonucleotide-conjugated secondary antibodies, followed by DNA amplification in the presence of NaveniFlex Cell Buffer 2 Red, was performed according to the protocol. Finally, nuclei and cytoplasm were counterstained using 4′,6-diamidino-2-phenylindole (DAPI, Sigma-Aldrich, Merck, Darmstadt, GERMANY) and phalloidin-FITC (Sigma-Aldrich, Merck, Darmstadt, GERMANY), for 5 or 20 min at RT, respectively. Glass slides were mounted with a coverslip using the Fluoromount™ Aqueous Mounting Medium (Sigma-Aldrich, Merck, Darmstadt, GERMANY) and then imaged with a Nikon Eclipse T*i* inverted microscope (Nikon Corporation, Tokyo, JAPAN) with the Spinning Disk, using 60× magnification. The red spots, representing a single protein–protein interaction, were counted on raw images by automatic particle analysis (ImageJ software, NIH, Bethesda, MA, USA), and data were reported as a function of cell number calculated in each field of view. For each experiment, three representative fields of vision were reported.

### Soft agar colony formation assay

Anchorage-independent cell growth was studied in FTC-133 cells by performing a soft agar colony formation assay, after cell preconditioning with IDO1 catalytic inhibitors or iDeg-1 for 24 h. Firstly, the bottom of a 6-well plate was precoated with a mix (1:1 ratio) of 1% agarose solution and 2X complete DMEM/F12 medium (1.5 mL/well). Then, preconditioned cells were resuspended in 2X complete DMEM/F12 medium (containing IDO1 catalytic inhibitors, iDeg-1, or vehicle) and 0.6% agarose solution (1:1 ratio) and plated (5 × 10^3^ cells/well) on the precoated 6-well plate (1.5 mL/well). The stimulus renewal was performed once a week. After 21 days, colonies were imaged using a Nikon Eclipse T*i* inverted microscope (Nikon Corporation, Tokyo, JAPAN) with 10× magnification. The size of the colonies was measured using the NIS-Elements AR Software (Nikon Corporation, Tokyo, JAPAN), considering the colonies in all fields, visualized from left to right, in the upper, middle, and lower regions of the whole well.

### Scratch wound healing assay

For the scratch wound healing assay, FTC-133 cells were seeded at 0.2 × 10^6^ cells/mL in 2 mL in a 6-well plate and cultured overnight to reach approximately over 80% confluence. After 24 h of preconditioning with IDO1 catalytic inhibitors, iDeg-1, or vehicle, or after 48 h of siRNA transfection, a 10-µL sterile pipette tip was used to make a scratch line on the monolayer of confluent cells at the bottom of the well. Cells were washed twice to remove cellular debris, re-exposed to stimuli or vehicle as appropriate, and incubated at 37°C in a humidified 5% CO_2_ incubator for the indicated time. Over this incubation time, the wound healings were continuously observed using a Nikon Eclipse T*i* inverted microscope (Nikon Corporation, Tokyo, JAPAN) with 10× magnification, and pictures were acquired at the indicated time points. The area of the wound healing was determined using the MRI Wound Healing tool (ImageJ software, NIH, Bethesda, MA, USA), and data were reported as a percentage of the wound closure with respect to time 0.

### Transwell migration assay

Transwell migration assay in FTC-133 cells was performed using 8 μm polycarbonate membrane Transwell^®^ inserts (Corning, NY, USA) in a 24-well plate. Briefly, FTC-133 cells were resuspended at 0.5 × 10^6^ cells/mL in culture medium without FCS and treated with IDO1 catalytic inhibitors, iDeg-1, or vehicle. In the case of knockdown experiments, FTC-133 cells were transfected with siRNA 48 h before the transwell migration assay. Then, 150 µL of cell suspension were seeded into the upper chamber of the insert, over a lower compartment containing either 600 µL of complete culture medium (10% FCS as chemoattractant) or 600 µL of culture medium without FCS as a negative control. After 48 h of incubation at 37°C, transwell inserts were washed twice with PBS, and cells on the upper surface were removed by moistened cotton swabs. Cells on the lower surface were fixed with 4% PFA for 15 min at RT and then stained with 0.1% crystal violet (Sigma-Aldrich, Merck, Darmstadt, GERMANY) in bidistilled water for 30 min at RT. After washing with PBS, inserts were air-dried and then imaged using an EVOS M5000 Imaging System (Thermo Fisher Scientific, Waltham, MA, USA) with 10× magnification. Cell migration was analyzed on raw images by automatic particle analysis (ImageJ software, NIH) and was reported as the percentage of cell-covered area, calculated in each field of view. For each experiment, three representative fields of vision were reported.

### Statistical analysis

All analyses were performed using Prism version 10.3.1 (GraphPad software). Data were shown as mean ± SD of at least three independent experiments. Data were analyzed by one-way or two-way ANOVA followed by *post-hoc* Bonferroni’s test. A p-value less than 0.05 was considered significant. The half-life (t_1/2_) and the degradation speed (K) of the IDO1 protein were calculated by non-linear regression analysis of raw data using the “one-phase decay” equation.

## Results

### The catalytic inhibitors of IDO1 increase the protein expression in tumor cells

**Figure 1:**
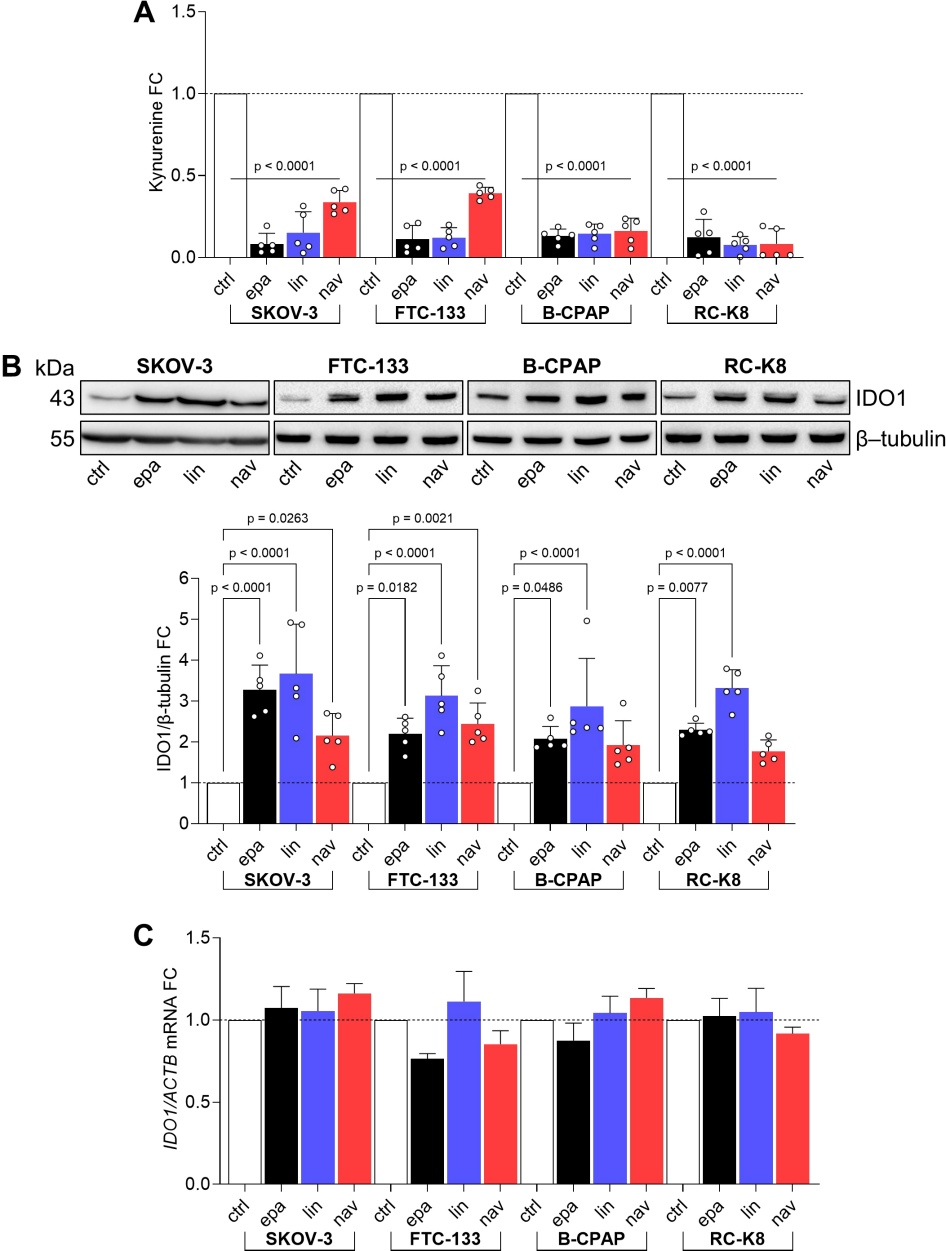
IDO1 catalytic inhibitors increase IDO1 protein expression in human tumor cell lines. **(A)** Kynurenine released by SKOV-3, FTC-133, B-CPAP, and RC-K8 cells treated with EPA, LIN, and NAV (1 µM) for 24 h Vehicle-treated cells were used as a control (ctrl). Results are shown as kynurenine fold change (FC) of treated *versus* respective ctrl cells (dotted line, 1-fold). **(B)** IDO1 protein expression in SKOV-3, FTC-133, B-CPAP, and RC-K8 cells treated as in **(A)**. β-tubulin expression was used as a normalizer. One representative immunoblot of five is shown. The IDO1/β-tubulin ratio of scanning densitometry analysis is reported as FC of treated versus respective ctrl cells (dotted line, 1-fold). **(C)** Real-time PCR analysis of *IDO1* transcript in SKOV-3, FTC-133, B-CPAP, and RC-K8 cells treated as in **(A)**, normalized to the expression of *ACTB* and reported as relative to the results in the respective ctrl cells (dotted line, 1-fold). Data in **(A–C)** are mean ± SD of independent experiments (**A**, **B**: *N* = 5; **C**: *N* = 3) and were analyzed by one-way ANOVA followed by *post hoc* Bonferroni’s test. Statistical significance shown in **(A)** is representative of all comparisons (EPA, LIN, or NAV *versus* ctrl).

Based on our previous data describing the effect of epacadostat (hereafter EPA) in promoting the non-enzymatic function of IDO1 in both murine plasmacytoid DCs and in the SKOV-3 cell line (20, 23), we extended our screening to two further catalytic inhibitors of IDO1, namely, navoximod and linrodostat (hereafter NAV and LIN, respectively), in various human cancer cell lines endogenously expressing IDO1 protein. All three inhibitors reached advanced clinical phases and inhibit the IDO1 enzyme by a different mechanism of action (9). Specifically, EPA and NAV belong to the first-generation inhibitors that bind the holo-enzyme (i.e., containing the heme cofactor) and inhibit IDO1 by a competitive and non-competitive mechanism, respectively. LIN is a second-generation IDO1 inhibitor that binds and stabilizes the heme-free conformation of IDO1, also named a heme-displacing inhibitor (24). The three different IDO1 inhibitors were tested in different human cancer cell lines that share an endogenous and constitutive expression of the IDO1 enzyme, catalyzing the conversion of Trp to Kyn. Specifically, SKOV-3, FTC-133, and B-CPAP cells from solid human tumors and RC-K8 from hematological cancers were used. First, we evaluated the IDO1 catalytic inhibition by EPA, LIN, and NAV in the cancer cell lines after exposure to 1 μM of inhibitors for 24 h, by measuring Kyn levels in the culture supernatants. All three inhibitors significantly reduced Kyn levels in the cancer cell line supernatants with a fold change (FC) <0.5 versus the control cells (**Figure 1A**), indicating the efficacy of the IDO1 catalytic inhibitors in all the tested cancer cell lines. We also analyzed in the same samples the IDO1 protein level by Western blot, and surprisingly, we observed an increased level of IDO1 protein in all the cancer cell lines exposed to EPA, LIN, and NAV (**Figure 1B**, upper panel). The quantitative analyses of the normalized IDO1 protein showed a variable protein increase in response to the type of inhibitor, depending on the cancer cell line. However, the IDO1 protein level was increased in most of the inhibitor-treated cells with an FC >2 compared to the control represented by the basal expression of the IDO1 protein in the cancer cell line (**Figure 1B**, lower panel). As an autocrine AhR-IL-6-STAT3 signaling loop sustained the expression of IDO1 in tumor cells fuelled by Kyn that acts as an AhR agonist (25), we analyzed *IDO1* gene transcription in the cancer cell lines exposed to the three catalytic inhibitors, and we observed that none of the inhibitors significantly induced the *IDO1* gene expression in any of the cell lines (**Figure 1C**), excluding a transcriptional mechanism involved in the increased IDO1 protein expression by catalytic inhibitors.

Overall, these data suggest an additional “on-target” effect of IDO1 catalytic inhibitors in the different tumor cell lines. A catalytically inactive conformation of the IDO1 protein is increased by the inhibitors, through a mechanism that does not involve gene transcription and in a manner independent of their mechanism of inhibition and tumor type.

### The catalytic inhibitors of IDO1 prolong the protein half-life in the FTC-133 cell line

**Figure 2:**
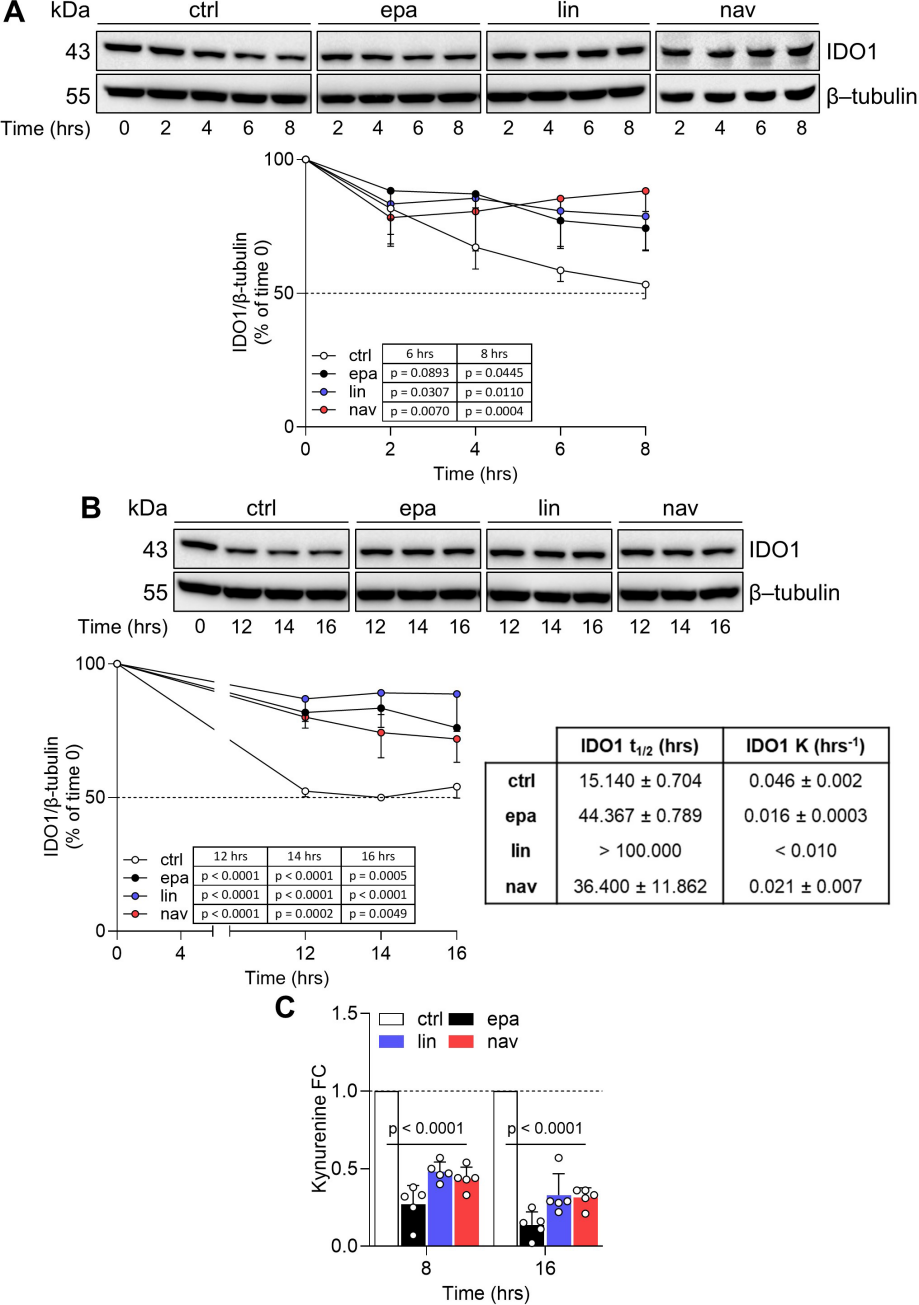
IDO1 catalytic inhibitors prolong the IDO1 protein half-life in FTC-133 cells. **(A, B)** Cycloheximide chase assay followed by immunoblot analysis of IDO1 protein expression in FTC-133 cells pretreated with cycloheximide (50 μg/mL) for 1 h (as time 0) and then exposed to EPA, LIN, and NAV (1 µM) for a short (up to 8 h) **(A)** or long (up to 16 h) **(B)** kinetics. For each time point, vehicle-treated cells were used as a control (ctrl), whereas β-tubulin expression was used as a normalizer. One immunoblot representative of three is shown. The exponential decay regression analysis of the IDO1/β-tubulin protein ratio is expressed as a percentage (%) of time 0 (time 0 = 100%; dotted line, 50%). The half-life (*t*_1/2_, in hours) and the degradation speed (K, in hours^−1^) of IDO1 protein are reported as mean ± SD and shown in the table (**B**, lower right panel). **(C)** Kynurenine released by FTC-133 cells treated as in **(A, B)**, measured at the last time points of the kinetics. Results are shown as kynurenine FC of treated *versus* respective ctrl cells (dotted line, 1-fold). Data in **(A–C)** are mean ± SD of independent experiments (**A**, **B**: *N* = 3; **C**: *N* = 5) and were analyzed by two-way ANOVA followed by *post-hoc* Bonferroni’s test. Statistical significance shown in **(C)** is representative of all comparisons (EPA, LIN, and NAV *versus* the respective ctrl).

Previous data demonstrated that the expression of IDO1 in FTC-133 cells plays a relevant role in the immunosuppression of the TME (26) and that the Kyn-driven activation of AhR might play an oncogenic function (27). Moreover, FTC-133 cells express the highest level of *IDO1* among thyroid cancer lines (**Supplementary Figure 1**). We thus narrowed our investigational field to the thyroid cancer line FTC-133 after exposure to the three catalytic inhibitors, to deep inside the post-translational mechanism by which the IDO1 catalytic inhibitors increase the protein level in the tumor cells. To this purpose, we performed a cycloheximide chase assay followed by immunoblotting to analyze IDO1 protein turnover in FTC-133 cells when exposed to EPA, LIN, and NAV. After blocking the *de novo* synthesis of IDO1 by cycloheximide, we monitored the IDO1 protein level over 16 h in FTC-133 cells. Surprisingly, we detected a sustained level of IDO1 protein in the inhibitor-treated FTC-133 cells compared to control cells over a time period ranging from 4 h (**Figure 2A**) up to 16 h (**Figure 2B**). The quantitative analyses of the normalized protein expression confirmed that the turnover of the IDO1 protein is significantly slower in FTC-133 cells treated with EPA, LIN, and NAV as compared to the control cells. Specifically, the treatment of the cancer cells with all three types of catalytic inhibitors increased the IDO1’s protein half-life with a *t*_1/2_ >44.367 ± 0.789 h, >100 h, and >36.400 ± 11.862 h, for EPA, LIN, and NAV, respectively, as compared to the physiologic turnover of IDO1 in FTC-133 cells (*t*_1/2_ > 15.140 ± 0.704 h) (**Figure 2B**, right lower panel). Although the resulting IDO1 protein level is increased by the catalytic inhibitors, its enzymatic activity is inhibited, as indicated by the significantly reduced Kyn levels detected in the supernatants of inhibitor-treated cells compared to the control (**Figure 2C**). Interestingly, an overlapping effect was observed in the human ovarian cancer cell line SKOV-3, where EPA (20), LIN, and NAV also prolonged the half-life of the IDO1 protein while simultaneously inhibiting its catalytic activity (**Supplementary Figures 2A, B**).

Altogether, the data indicate that IDO1 catalytic inhibitors sustained the expression of a non-enzymatic conformation of IDO1 in the FTC-133 cancer cell line through a post-translational mechanism that prolongs its protein half-life.

### Non-enzymatic IDO1 acts as a signaling molecule in the FTC-133 cell line

**Figure 3:**
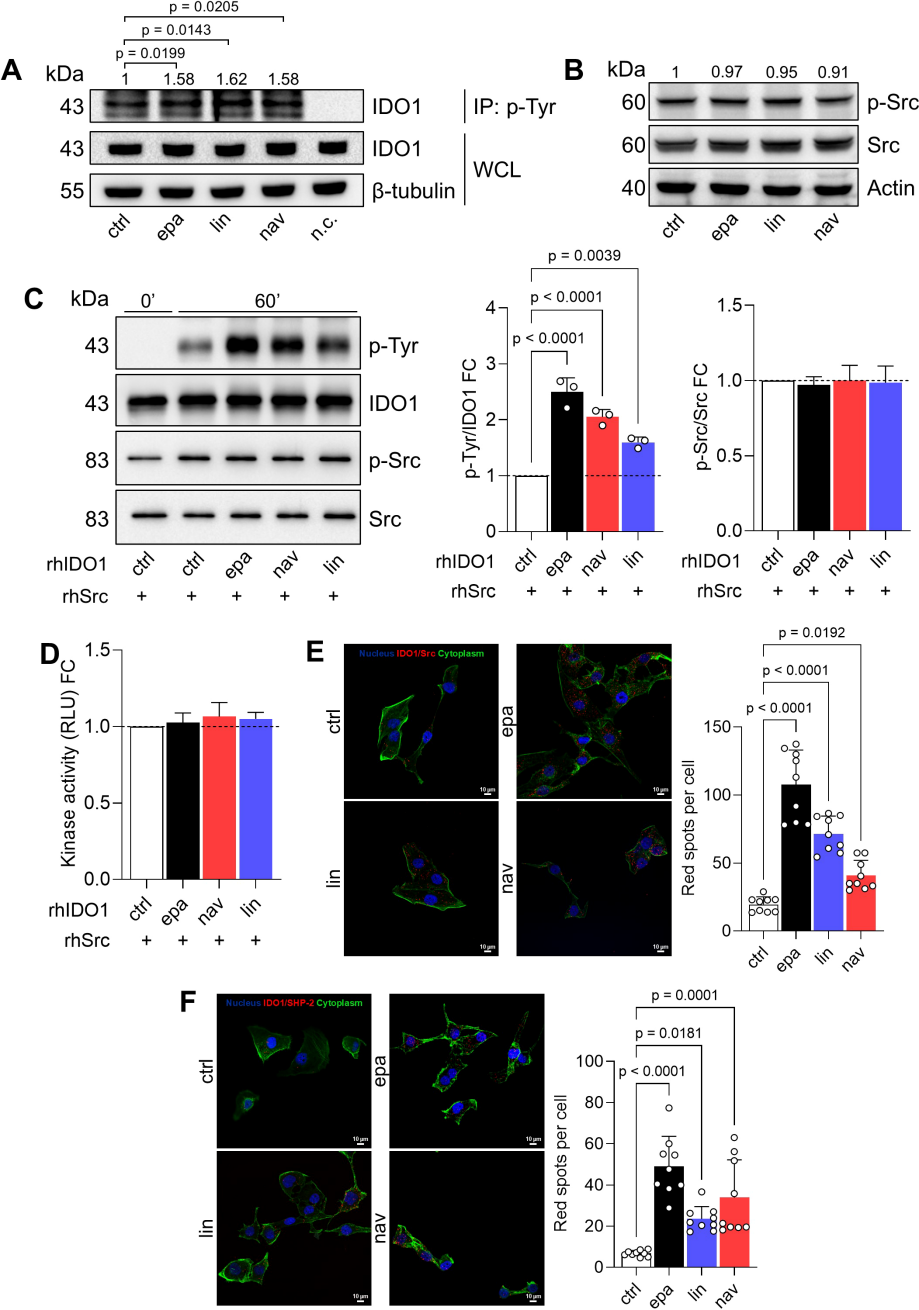
IDO1 catalytic inhibitors enhance the non-enzymatic function of IDO1 in FTC-133 cells. **(A)** p-Tyr immunoprecipitation (IP) and IDO1 immunoblot analysis in FTC-133 cells treated with EPA, LIN, and NAV (1 µM) for 4 h Vehicle-treated cells were used as control (ctrl), whereas WCLs were used as controls of input protein expression, and a negative control (n.c.; i.e., only resin) was included. **(B)** Phosphorylated Src (p-Src) and total Src immunoblot analysis of FTC-133 cells treated as in **(A)**. In **(A, B)** β-tubulin or actin were used as normalizers and quantitative data from densitometric analysis (pTyr-IDO1 or pSrc/Src ratios, respectively) are reported as FC of treated *versus* ctrl cells (1-fold), above the corresponding bands. **(C)** Tyr-phosphorylation of rhIDO1 incubated with rhSrc in the presence of ATP (100 µM) for 60 min. rhIDO1 was preconditioned with EPA, NAV, and LIN (10 μM) for 30 min prior to rhSrc exposure, while vehicle-preconditioned rhIDO1 was used as a ctrl. The p-Tyr/IDO1 ratio and the p-Src/Src ratio were calculated by densitometric analysis and reported as FC of EPA/LIN/NAV-conditioned samples *versus* the ctrl sample (60 min; dotted line, 1-fold). **(D)** Kinase activity of rhSrc incubated as in **(C)** and reported as FC of relative light unit (RLU) measured in EPA/LIN/NAV-conditioned samples *versus* the ctrl sample (dotted line, 1-fold). **(E)** *In situ* PLA between IDO1 and Src performed in FTC-133 cells treated with EPA, LIN, and NAV (1 μM) for 1 h **(F)** *In situ* PLA between IDO1 and SHP-2 performed in FTC-133 cells treated with EPA, LIN, and NAV (1 μM) for 16 h In **(E, F)** vehicle-treated cells were used as ctrl. For each condition, a representative field of view is shown (60× magnification; scale bar: 10 µm) and the number of red spots, indicating the single IDO1/Src **(E)** or IDO1/SHP-2 **(F)** interaction, is reported as a function of cell number in each field of view. For **(A–C)** one representative immunoblot of three is shown. Data in **(A–F)** are mean ± SD of independent experiments (*N* = 3) and were analyzed by one-way ANOVA followed by *post-hoc* Bonferroni’s test.

As we observed the stabilization of a non-enzymatic conformation of IDO1 by the three different catalytic inhibitors in the FTC-133 cell line, we explored the signaling activity of IDO1 in these cells. Interestingly, the exposure of FTC-133 cells to EPA, LIN, and NAV promoted the tyrosine-phosphorylation of IDO1, as shown by the increased level of tyrosine-phosphorylated IDO1 in inhibitor-treated FTC-133 cells as compared to control cells (**Figure 3A**). The non-receptor tyrosine kinase Src has previously been shown to phosphorylate the IDO1 protein, enabling it to act as a signaling molecule (28, 29). We thus explored the expression and the activation of Src kinase in the FTC-133 cell line through the analysis of phosphorylated human Src at the tyrosine Y419 as a surrogate biomarker of kinase activation (28). The kinase Src was constitutively expressed and activated in FTC-133 cells, and the level of phosphorylated Src did not change in the presence of IDO1 catalytic inhibitors, excluding an off-target activation of Src by the compounds (**Figure 3B**). As a proof of concept, we investigated the molecular relationship between Src kinase, IDO1, and its catalytic inhibitors in a cleaner cell-free system, using the recombinant human enzymes (namely, rhSrc and rhIDO1) and the catalytic inhibitors. Specifically, we evaluated the phosphorylation of IDO1 by Src kinase activity in response to the compounds. Notably, we found that the preconditioning of rhIDO1 with EPA, LIN, and NAV significantly increased the level of phosphorylated IDO1 after incubation with rhSrc for 60 min at 25°C (**Figure 3C**), but it did not change the level of phosphorylated Src (**Figure 3C**) or its kinase activity (**Figure 3D**), confirming the previous observation in the FTC-133 cell line (**Figures 3A, B**).

We thus hypothesized that IDO1 catalytic inhibitors stabilize a conformation of IDO1 more prone to interact with and be phosphorylated by Src kinase, as also demonstrated in (30). We corroborated the biological relevance of our data collected in both the cell and recombinant systems by exploring the IDO1/Src interaction in FTC-133 cells after incubation with the IDO1 catalytic inhibitors. Interestingly, we observed that the constitutive IDO1/Src association in FTC-133 cells significantly increased in the presence of EPA, LIN, and NAV (**Figure 3E**, **Supplementary Figure 3A**), in line with the increase of phosphorylated IDO1 in inhibitor-treated FTC-133 cells (**Figure 3A**). Of importance, tyrosine-phosphorylation makes IDO1 capable of interacting with SHP-2 phosphatase, a known oncogene (31) previously observed in association with IDO1 in SKOV-3 cells in response to epacadostat (20, 31). Analogously, the exposure of FTC-133 cells to EPA, LIN, and NAV significantly increased the red spot number per cell, representing the number of IDO1/SHP-2 interactions, as compared to the control (**Figure 3F**, **Supplementary Figure 3B**). Overall, these data confirm that EPA, LIN, and NAV shape a non-enzymatic conformation of IDO1 that can be phosphorylated by Src kinase and interact with downstream partners such as SHP-2 phosphatase. Thus, IDO1 acts as a signaling molecule in the tumor cells in response to the catalytic inhibitors.

### Pro-tumorigenic side effects of IDO1 catalytic inhibitors in the FTC-133 cell line

**Figure 4:**
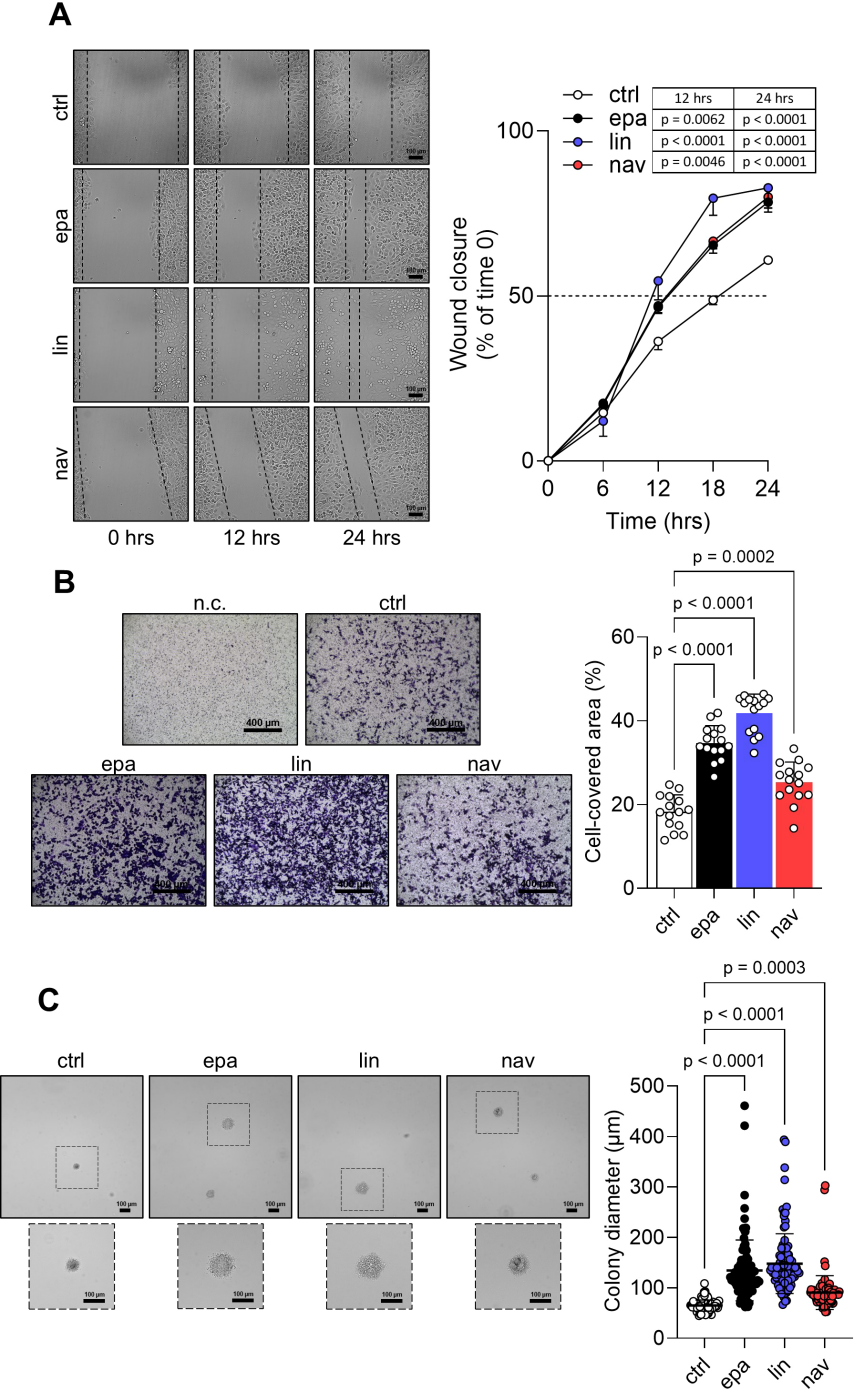
Pro-tumorigenic effects of IDO1 catalytic inhibitors in FTC-133 cells. **(A)** Analysis of the wound closure (black dotted lines) on FTC-133 cells in the presence of EPA, LIN, and NAV (1 µM) or vehicle alone (ctrl) over time. For each reported time point, one representative image of three is shown (10× magnification; scale bar: 100 µm), and data are reported as wound closure percentage (%) of respective time 0 (time 0 = 0%; dotted line, 50%). **(B)** Transwell migration assay performed on FTC-133 cells exposed to EPA, LIN, and NAV (1 µM) in serum-free medium. Vehicle-treated cells were used as ctrl, while vehicle-treated cells cultured over serum-free medium were included as a negative ctrl (n.c.). For each condition, a representative field of view is shown (10× magnification; scale bar: 400 µm), and cell migration is reported as percentage (%) of cell-covered area, calculated in each field of view. **(C)** Soft agar colony formation assay in FTC-133 cells exposed to EPA, LIN, and NAV (1 µM) or vehicle alone (ctrl) once a week. For each condition, the colony size (µm) of 100 representative colonies is reported, whereas one representative image of three is shown (10× magnification; scale bar: 100 µm). Data in **(A–C)** are mean ± SD of independent experiments (**A**, **C**: *N* = 3; **B**: *N* = 5). Data in **(A**) and **(B, C)** were analyzed by two-way or one-way ANOVA followed by *post-hoc* Bonferroni’s test, respectively.

As the interaction of the well-established oncogene SHP-2 with phosphorylated IDO1 increased in FTC-133 cells in response to the catalytic inhibitors, we explored the functional effects triggered by IDO1/SHP-2 complexes in the tumor cells. First, we monitored the migratory capacity of inhibitor-treated FTC-133 cells over 24 h by the scratch assay. Interestingly, we observed that the exposure of FTC-133 cells to EPA, LIN, and NAV promotes a faster migration of the cells compared to the control. After only 12 h of incubation, the rate of wound closure was significantly higher in FTC-133 cells treated with inhibitors compared to the control group, and this difference remained significant for up to 24 h (**Figure 4A**). Analogously, the quantification of the migrated cells by the Transwell assay revealed a significantly higher percentage for inhibitor-treated FTC-133 cells compared to the control (**Figure 4B**). Moreover, the anchorage-independent proliferation of FTC-133 cells was also enhanced by EPA, LIN, and NAV, which significantly increased the growth in a semisolid matrix, as indicated by the 2-fold (for EPA and LIN) and 1.5-fold (for NAV) increases in colony size compared to control cells (**Figure 4C**).

Altogether, these data demonstrate that IDO1 catalytic inhibitors promote in the FTC-133 tumor cells an intrinsic side effect that enables the cells to migrate and proliferate faster than the control cells, resulting in a pro-tumorigenic effect.

### IDO1 protein degradation abrogates the pro-tumorigenic effects in the FTC-133 cell line

**Figure 5:**
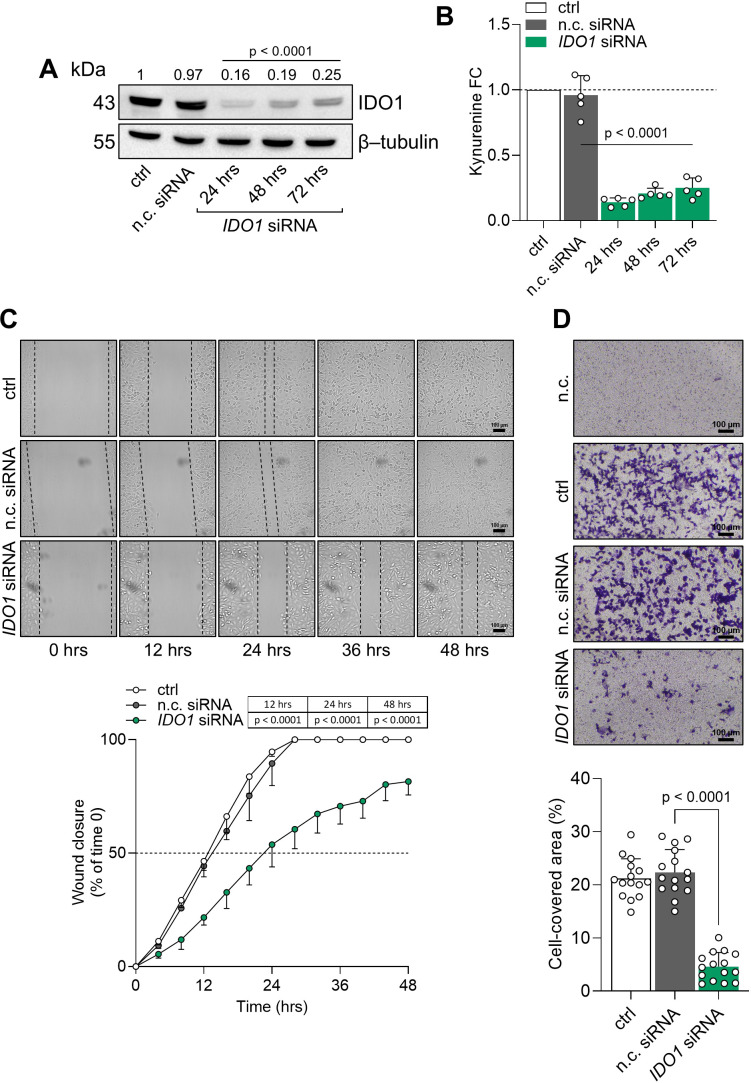
IDO1 protein knockdown abrogates the pro-tumorigenic phenotype of FTC-133 cells. **(A)** Immunoblot analysis of IDO1 protein expression in FTC-133 cells transfected with IDO1-specific (*IDO1* siRNA) or negative control siRNAs (n.c. siRNA). Untreated cells were used as a control (ctrl), whereas β-tubulin expression was used as a normalizer. One representative immunoblot of five is shown. The IDO1/β-tubulin protein ratio of scanning densitometry analysis is reported as FC of siRNA-transfected *versus* respective ctrl cells (1-fold), above the corresponding bands. **(B)** Kynurenine released by FTC-133 cells treated as in **(A)**. Results are shown as kynurenine FC of siRNA-transfected *versus* respective ctrl cells (dotted line, 1-fold). In **(A, B)**, representative data at 72 h were shown for the ctrl and n.c. siRNA samples. **(C)** Analysis of the wound closure (black dotted lines) in FTC-133 cells after 48 h of transfection with *IDO1* or n.c. siRNAs, over time. Untreated cells were used as ctrl. For each reported time point, one representative image of three is shown (10× magnification; scale bar: 100 µm), and data are reported as wound closure percentage (%) of respective time 0 (time 0 = 0%; dotted line, 50%). **(D)** Transwell migration assay performed on FTC-133 cells in serum-free medium, after 48 h of transfection with *IDO1* or n.c. siRNAs. Untreated cells were used as a ctrl, while untreated cells cultured over serum-free medium were included as a negative control (n.c.). For each condition, a representative field of view is shown (10× magnification; scale bar: 100 µm), and cell migration is reported as percentage (%) of cell-covered area, calculated in each field of view. Data in **(A, B, D)** and **(C)** are mean ± SD of independent experiments (**A**, **B**, **D**: *N* = 5; **C**: *N* = 3). Data in **(A-D)** were analyzed by one-way or two-way ANOVA followed by *post hoc* Bonferroni’s test, respectively, comparing *IDO1* siRNA *versus* n.c. siRNA. Statistical significance shown in **(A, B)** is representative of all comparisons (*IDO1* siRNA at each time point *versus* n.c. siRNA).

**Figure 6:**
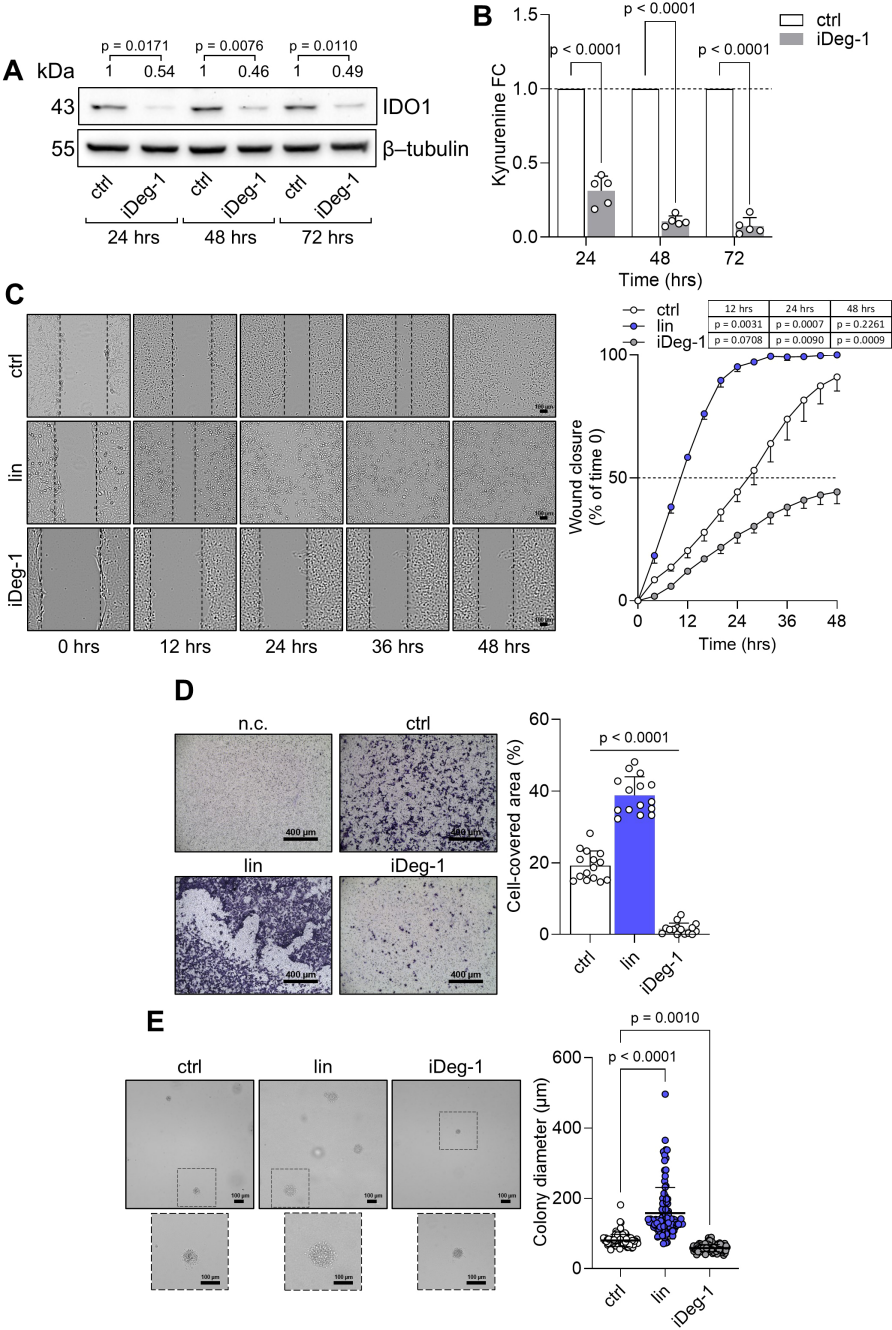
IDO1 protein degradation abrogates the pro-tumorigenic phenotype of FTC-133 cells. **(A)** Immunoblot analysis of IDO1 protein expression in FTC-133 cells exposed to iDeg-1 (10 µM) or vehicle alone (ctrl). β-tubulin expression was used as a normalizer. One representative immunoblot of five is shown. The IDO1/β-tubulin protein ratio of scanning densitometry analysis is reported as FC of treated *versus* respective ctrl cells (1-fold), above the corresponding bands. **(B)** Kynurenine released by FTC-133 cells treated as in **(A)**. Results are shown as kynurenine FC of treated *versus* respective ctrl cells (dotted line, 1-fold). **(C)** Analysis of the wound closure (black dotted lines) in FTC-133 cells in the presence of LIN (1 µM), iDeg-1 (10 µM), or vehicle alone (ctrl) over time. For each reported time point, one representative image of three is shown (10× magnification; scale bar: 100 µm), and data are reported as wound closure percentage (%) of respective time 0 (time 0 = 0%; dotted line, 50%). **(D)** Transwell migration assay performed on FTC-133 cells exposed to LIN (1 µM) or iDeg-1 (10 µM) in serum-free medium. Vehicle-treated cells were used as a control, while vehicle-treated cells cultured over serum-free medium were included as negative control (n.c.). For each condition, a representative field of view is shown (10× magnification; scale bar: 400 µm), and cell migration is reported as percentage (%) of cell-covered area, calculated in each field of view. **(E)** Soft agar colony formation assay in FTC-133 cells exposed to LIN (1 µM), iDeg-1 (10 µM), or vehicle alone once a week. For each condition, the colony size (µm) of 100 representative colonies is reported, whereas one representative image of three is shown (10× magnification; scale bar: 100 µm). Data in **(A–E)** are mean ± SD of independent experiments (**A**, **B**, **D**: *N* = 5; **C**, **E**: *N* = 3). Data in **(A–C)** and **(D, E)** were analyzed by two-way or one-way ANOVA followed by *post-hoc* Bonferroni’s test, respectively. Statistical significance shown in **(D)** is representative of all comparisons (LIN or iDeg-1 *versus* ctrl).

The intrinsic “on-target” effect of IDO1 catalytic inhibitors in FTC-133 cells is mediated by the stabilization of the IDO1 protein in a non-enzymatic conformation suitable for mediating signal transduction in the tumor cells that results in an adverse effect. By contrast, the knockdown of endogenous IDO1 in FTC-133 cells by specific siRNA abrogated the protein expression and significantly decreased the level of Kyn production compared to the n.c. siRNA-treated cells and untreated cells after 24 h to 72 h (**Figures 5A, B**). Interestingly, IDO1 knockdown significantly slowed the basal migratory ability of FTC-133 cells that could not close the wound over 48 h compared to the n.c. siRNA-treated cells that reached 100% closure before 36 h, overlapping the migration of untreated FTC-133 cells (**Figure 5C**). Moreover, the percentage of migrated IDO1-silenced FTC-133 dramatically decreased compared to the negative controls (**Figure 5D**). Overall, IDO1 knockdown in FTC-133 cells appears to be directly responsible for a less aggressive phenotype and supports the FTC-133 phenotype when the cells are exposed to IDO1 catalytic inhibitors. Thus, the mere catalytic inhibition of the IDO1 enzyme results in ineffectiveness for targeting the tumor growth in the TME because of the additional signaling function of the IDO1 protein in the tumor. To counteract the dual effect of IDO1 in FTC-133 cells, we employed a degrader protein strategy. For this purpose, we resorted to iDeg-1, a small molecule selected for its biological and drug-like properties within a pseudo-natural product collection. Previous studies characterized iDeg-1 as both a catalytic inhibitor and a degrader of IDO1 (21). Firstly, we investigated the dual inhibitory effect of iDeg-1 in the IDO1-expressing FTC-133 cell line by monitoring both the protein expression and the enzymatic activity over 72 h of exposure to iDeg-1. Interestingly, iDeg-1 exerted a long-lasting inhibitory effect on IDO1, mediated by protein degradation and kynurenine production decrease. Compared with control cells, protein expression was significantly halved as early as 24 h, and this effect lasted up to 72 h (**Figure 6A**), while kynurenine levels dramatically decreased throughout the 72-h period (**Figure 6B**). We thus evaluated the functional effects of iDeg-1 in FTC-133 cells by comparing cell migration and proliferation of iDeg-1- and LIN-treated cells. Surprisingly, we observed opposite behaviors of iDeg-1 and LIN with respect to FTC-133 cell migration. As shown in **Figure 6C**, treatment with iDeg-1 significantly slowed wound closure that remained open and did not reach 50% closure after 48 h, compared with the control. In contrast, LIN-treated cells migrated faster than control and iDeg-1-exposed cells, achieving complete wound closure within 24 h. We also observed an overlapping behavior in the Transwell migration assay. The quantification of migrated FTC-133 cells showed a significant decrease in cells treated with iDeg-1 and an increase in cells treated with LIN compared to control cells (**Figure 6D**). Lastly, while LIN increased the ability of FTC-133 to grow in an anchorage-independent manner, the exposure to iDeg-1 impaired this behavior, as demonstrated by the significant reduction in the size of colonies formed by FTC-133 cells in semisolid media (**Figure 6E**).

Overall, these findings demonstrate that iDeg-1 effectively degrades IDO1 protein in FTC-133 cells and abrogates the pro-tumorigenic side effect mediated by the catalytic inhibitor LIN, suggesting that protein degradation, rather than enzymatic inhibition, is an effective strategy for targeting IDO1 in the TME.

## Discussion

**Figure 7:**
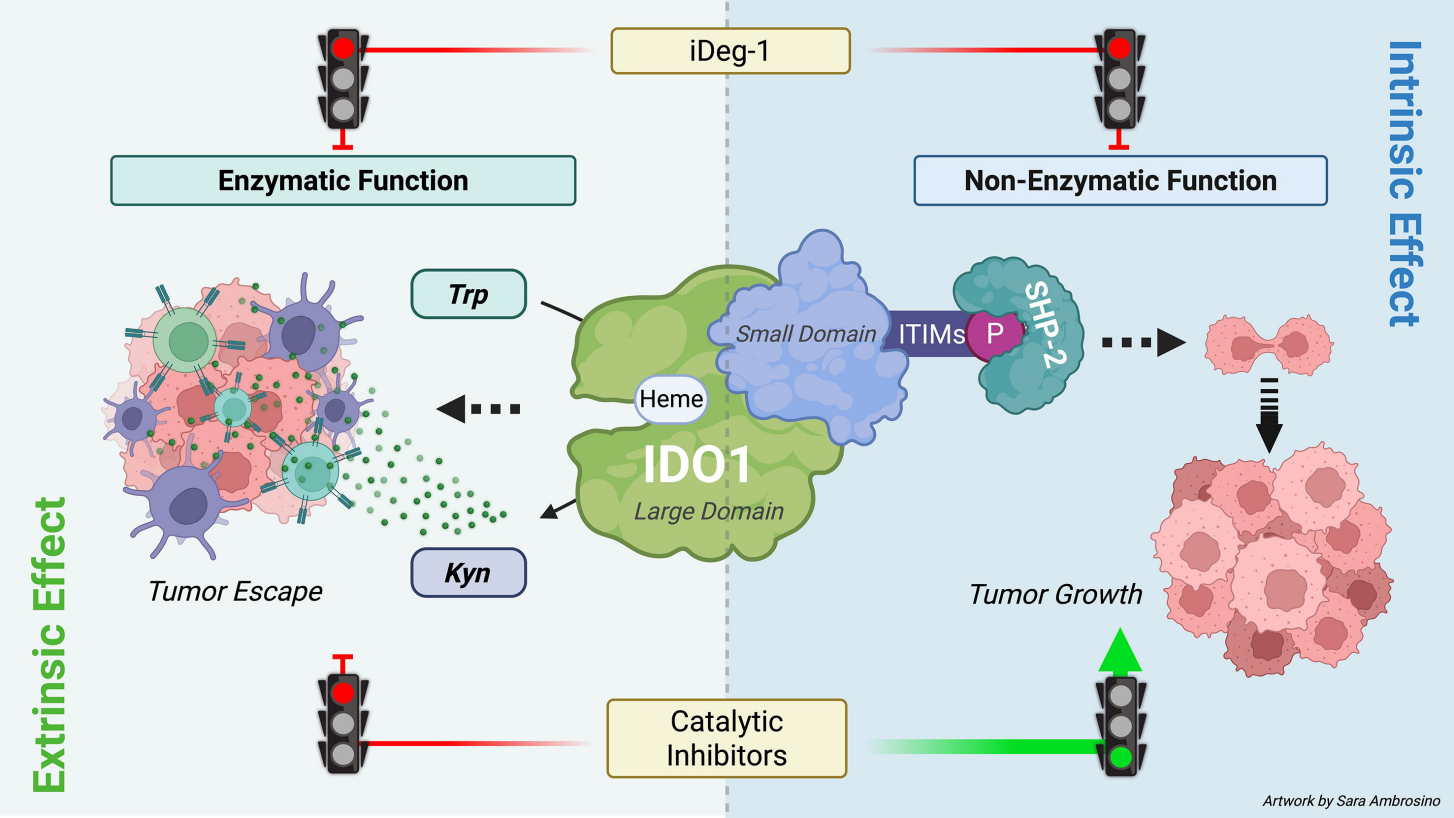
Dual role of IDO1 in the TME and therapeutic strategies. IDO1 exerts a dual role in the TME through its enzymatic and non-enzymatic functions. Its enzymatic function is mediated by the heme-containing catalytic site in the large domain, which converts tryptophan (Trp) into kynurenine (Kyn). Both Trp depletion and Kyn accumulation foster an immunosuppressive TME that facilitates tumor immune escape (extrinsic effect). The non-enzymatic function is triggered by phosphorylation of immunoreceptor tyrosine-based inhibitory motifs (ITIMs), which recruit SHP-2 and activate downstream signaling within tumor cells, thereby promoting tumor growth (intrinsic effect). Conventional IDO1 catalytic inhibitors block only the enzymatic activity and may unexpectedly enhance ITIM-dependent signaling, limiting therapeutic efficacy. By contrast, iDeg-1 induces IDO1 degradation, abolishing both enzymatic and non-enzymatic functions, and thus achieves broader suppression within the TME (*image created with BioRender.com*).

IDO1 catalytic inhibitors were developed based on the idea of blocking the IDO1 enzyme, a recognized immunocheckpoint that allows tumor growth in the microenvironment. Different generations of inhibitors rapidly arrived in the clinical trials, where they highlighted an unexpected outcome. Although IDO1 catalytic inhibitors have demonstrated no apparent side effects, they failed to demonstrate therapeutic effectiveness as anticancer drugs. Efforts are underway toward the comprehension of this outcome and the suggestion of a more effective IDO1-based therapeutic approach. Recent and relevant preclinical studies paradoxically reported tumor-protective mechanisms as a consequence of IDO1 enzymatic inhibition. Specifically, IDO1 inhibition by epacadostat leads to an adverse protection of melanoma cells against IFN-γ-activated T cells, caused by Trp replenishment in the TME that prevents the downregulation of the transcription factor MITF responsible for melanoma cell survival (32). Furthermore, an unintended tumor-protective effect of the apo-IDO1 inhibitor B37 has been recently described through an intrinsic upregulation of the JAK2/STAT3 pathway in mouse colon cancer cells CT26 (33). In line with previous observations, the data in the current manuscript delve into the molecular mechanisms by which IDO1 inhibitors could cause an adverse tumor-protective effect. Regardless of their mechanism of inhibition, EPA, LIN, and NAV could stabilize and prolong the half-life of the non-enzymatic IDO1 protein conformation in various cancer types, including solid and hematological tumor cell lines. Consequently, the Kyn/Trp ratio decreases in the TME, disrupting the tolerogenic milieu and reinvigorating the antitumor immune response. Meanwhile, the non-enzymatic conformation of IDO1 inside the tumor cells still continues to trigger an unexpected pro-tumorigenic signaling, resulting in faster proliferation and migration of the tumor exposed to catalytic inhibitors. Indeed, conventional catalytic inhibitors exert a dual effect in the TME. First, they inhibit the enzymatic function of IDO1, preventing the conversion of Trp into immunoregulatory Kyn. As a consequence, both Trp depletion and Kyn accumulation are abrogated in the microenvironment. This “extrinsic” effect, driven by catalytic inhibition of IDO1, reshapes the TME and may disrupt IDO1-dependent immune evasion by the tumor. Second, IDO1 catalytic inhibitors display an unintended “side” effect. They stabilize the non-enzymatic conformation of IDO1, making it more prone to associate with the oncogene SHP-2 and to trigger an intracellular signaling pathway within the tumor cells. This “intrinsic effect” sustains the tumor proliferation (**Figure 7**). Thus, the intrinsic effect exerted by IDO1 catalytic inhibitors in the tumor cell represents an adverse “on-target” effect that promotes, rather than inhibits, the tumor growth. As a result, despite their expected anticancer effects, an unintended side effect of IDO1 catalytic inhibitors protects tumor survival and negates the therapeutic potential of these candidate molecules.

Due to IDO1’s moonlighting feature (12), the non-enzymatic conformation of the protein plays a relevant, cell-specific role that must be considered when developing candidate drugs that have to target IDO1 in the TME populated by both tumor and immune cells. Indeed, the non-enzymatic function of IDO1 promotes a tolerogenic, long-term phenotype in dendritic cells, which are capable of sustaining an immunosuppressive environment around the tumor (18). Differently, IDO1’s non-enzymatic activity in tumor cells activates a pro-tumorigenic signaling, enabling tumor proliferation (19, 20). Recent findings on the non-enzymatic function of IDO1 within the tumor cells underscore the urgent need to develop an effective approach capable of inhibiting IDO1’s dual function, including its enzymatic and signaling activities. Additionally, it is crucial to propose synergistic therapeutic associations by identifying novel molecular targets within the IDO1 signaling pathway. Our data demonstrated that the non-enzymatic IDO1 conformation could undergo tyrosine phosphorylation by Src kinase, which is constitutively activated in the FTC-133 tumor. This early molecular event in IDO1 signaling was potentiated when the IDO1 protein was exposed to EPA, LIN, and NAV, none of which affected Src kinase activity. This suggests that the catalytic inhibitors stabilize a conformation of IDO1 more prone to be phosphorylated by Src kinase, and promote the IDO1 signaling activity by interacting with the downstream phosphatase SHP-2. Both Src kinase and SHP-2 phosphatase have long been proposed as cancer drug targets, and many inhibitors have been developed as anticancer drugs (34, 35). These inhibitors could affect pro-tumorigenic IDO1 signaling, and synergistic combinations with IDO1 catalytic inhibitors should be tested in cancer immunotherapy. Additionally, recent findings highlighted the proteasome-associated deubiquitinating enzyme USP14, as an important target in colorectal cancer (CRC). The overexpression of USP14 in CRC stabilizes IDO1 protein and suppresses antitumor immunity (36). Based on our data, the USP14-mediated stabilization of the IDO1 protein could also promote its pro-tumorigenic signaling. Therefore, USP14 inhibition in association with IDO1 catalytic inhibitors may represent a promising strategy for CRC immunotherapy. Certainly, targeted IDO1 protein degradation could represent the most effective therapeutic option to inhibit both the enzymatic and non-enzymatic activity of IDO1. Several IDO1-directed PROTACs have recently been developed and characterized in preclinical models (37–39) as well as small molecules acting as monovalent degraders (21). Our data demonstrated that the small molecule iDeg-1 was able to degrade IDO1 protein and inhibit its enzymatic activity in FTC-133 cells with a long-lasting effect. Notably, iDeg-1 effectively inhibited FTC-133 proliferation and migration. These preliminary observations confirm the importance of blocking the dual activity of IDO1 in cancer.

Despite the strengths of our data that confirm the previous one (19, 20), we are aware that the study was conducted in immortalized cancer cell lines, and this could represent a limitation of the study. Nevertheless, this preliminary step was essential for identifying IDO1 molecular partners that contribute to the pro-tumorigenic signaling of IDO1, such as Src kinase and SHP-2 phosphatase, as well as for evaluating the pro-tumorigenic effects. To begin to address this limitation, we have expanded our observation to different human IDO1-expressing tumor cell lines, and we observed a similar IDO1 stabilization in response to three different catalytic inhibitors. Ideally, we will translate these preliminary findings into an *in vivo* model to confirm that non-enzymatic IDO1 plays a significant role in tumor survival mechanisms.

In conclusion, these insights into the moonlighting activity of IDO1 within the TME provide potential explanations for the clinical failures observed in the IDO1 inhibitor trials and suggest more effective IDO1 targeting in cancer to neutralize both its enzymatic and non-enzymatic functions in the TME.

## Data Availability

The raw data supporting the conclusions of this article will be made available by the authors, without undue reservation.
