# Supplementary material for: Adverse pro-tumorigenic effects of IDO1 catalytic inhibitors mediated by the non-enzymatic function of IDO1 in tumor cells
Source: Front Immunol. 2025 Nov 4;16:1680896. doi: 10.3389/fimmu.2025.1680896 (PMC12623407; doi:10.3389/fimmu.2025.1680896)
Supplement: Supplementary file 1 [file DataSheet1.pdf]

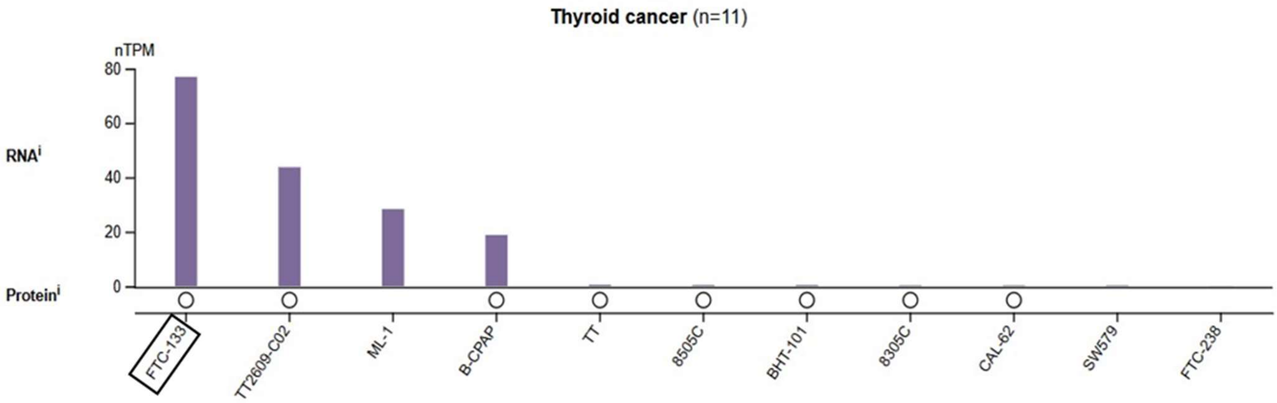

Supplementary Figure 1. IDO1 expression level in different thyroid cancer cell lines. Expression level of human IDO1 transcript (Ensembl: ENSG00000131203) in different human thyroid cancers, reported as normalized transcript per million (nTPM). The FTC-133 cell line is indicated by the black box. Image: Human Protein Atlas. Image/data available from https://www.proteinatlas.org/ENSG00000131203-IDO1/cell+line#thyroid_cancer.

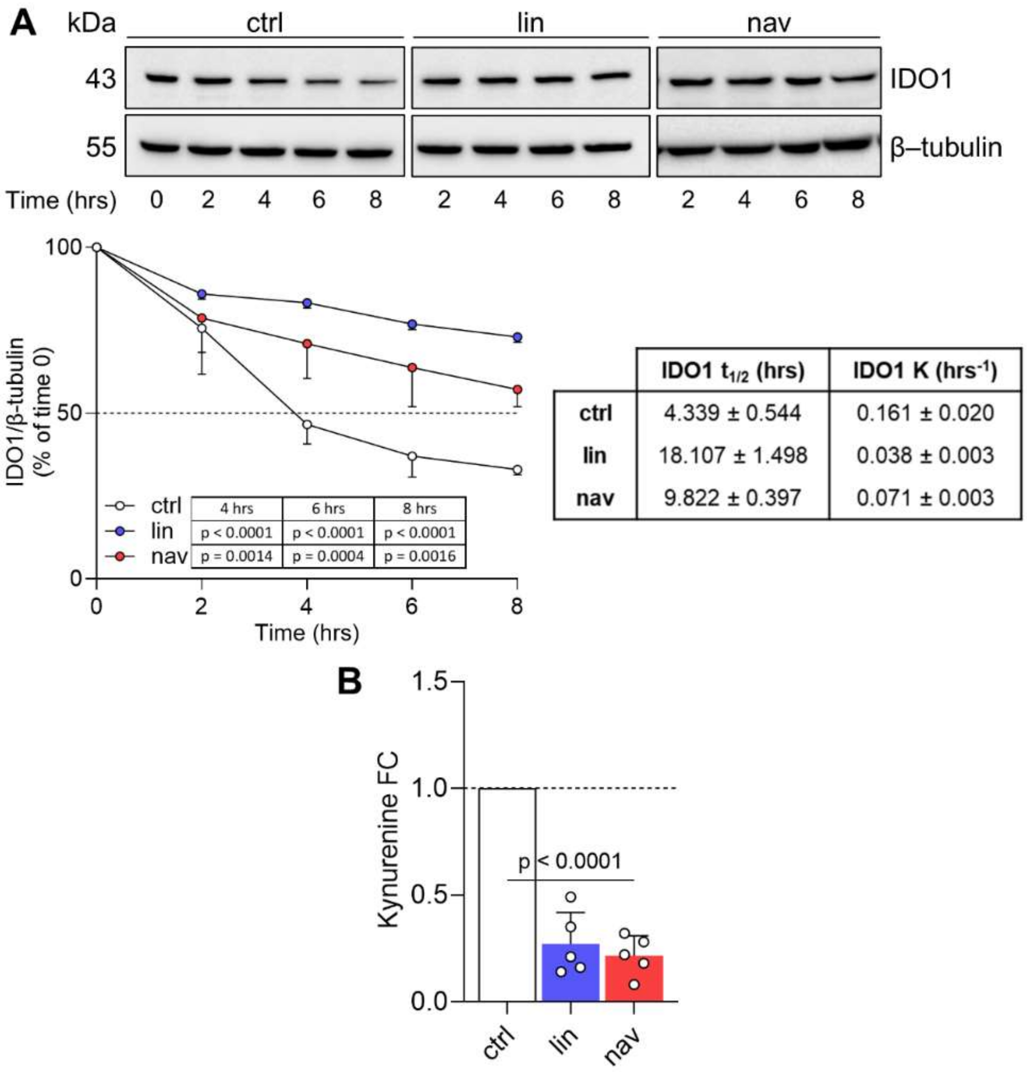

Supplementary Figure 2. IDO1 catalytic inhibitors prolong the IDO1 protein half-life in SKOV-3 cells. (**A**) Cycloheximide-chase assay followed by immunoblot analysis of IDO1 protein expression in lysates from SKOV-3 cells pre-treated with cycloheximide (50 µg/ml) for 1 hour (as time 0) and then exposed to LIN and NAV (1 µM) for the indicated time. For each time point, vehicle-treated cells were used as control (ctrl), whereas β-tubulin expression was used as a normalizer. One immunoblot representative of three is shown. The exponential decay regression analysis of the IDO1/β-tubulin protein ratio is expressed as percentage of time 0 (time 0 = 100%; dotted line, 50%). The half-life ($t_{1/2}$, in hrs) and the degradation speed (K, in hrs^{-1}) of IDO1 protein are reported as mean ± SD and shown in the table (**A**, lower right panel). (**B**) Kynurenine released by SKOV-3 cells treated as in (**A**), measured at the last time point of the kinetics. Results are shown as kynurenine FC of treated *versus* ctrl cells (dotted line, 1-fold). Data in (**A**, **B**) are mean ± SD of independent experiments (**A**: *N*=3; **B**: *N*=5). Data in (**A**) and (**B**) were analyzed by two-way or one-way ANOVA followed by *post-hoc* Bonferroni's test, respectively. Statistical significance shown in (**B**) is representative of all comparisons (LIN or NAV *versus* ctrl).

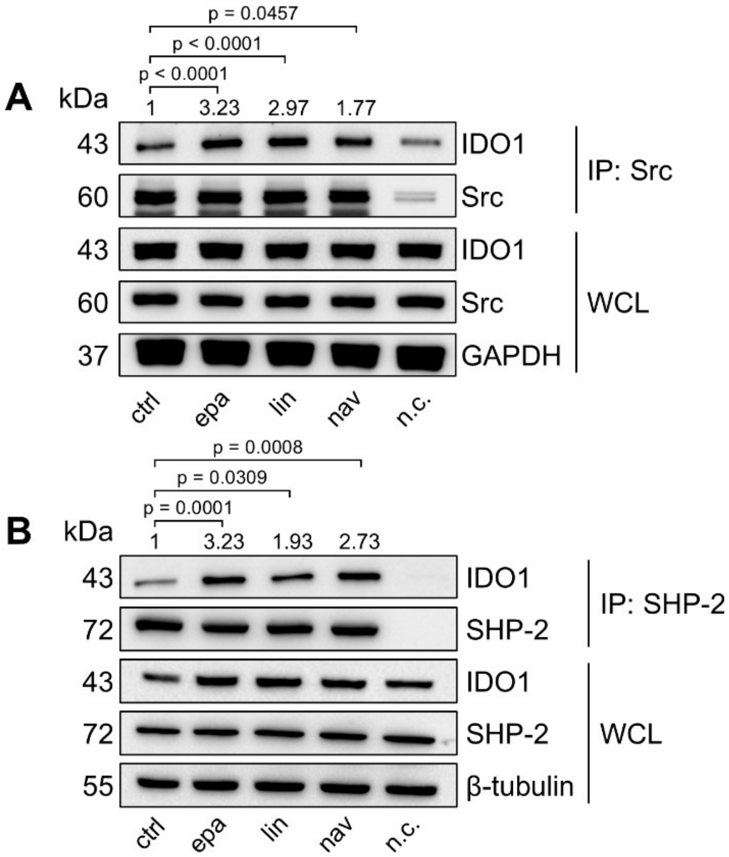

Supplementary Figure 3. IDO1 catalytic inhibitors promote the interaction of IDO1 with Src and SHP-2 in FTC-133 cells. (**A**) Src immunoprecipitation (IP) followed by Src and IDO1 immunoblot analysis performed in FTC-133 cells treated with EPA, LIN, and NAV (1 μM) for 1 hour. GAPDH expression was used as normalizer. (**B**) SHP-2 IP followed by SHP-2 and IDO1 immunoblot analysis performed in FTC-133 cells treated with EPA, LIN, and NAV (1 μM) for 16 hours. β-tubulin expression was used as normalizer. In (**A**, **B**), WCLs were used as controls of input protein expression and a negative control (n.c.; i.e., only resin) was included, whereas vehicle-treated cells were used as ctrl. In (**A**, **B**), one representative immunoblot of three is shown and quantitative data from densitometric analysis (IDO1/Src and IDO1/SHP-2 ratios, respectively) are reported as FC of treated *versus* ctrl cells (1-fold), above the corresponding bands. Data in (**A**, **B**) are mean ± SD of independent experiments (N=3) and were analyzed by one-way ANOVA followed by *post-hoc* Bonferroni's test.
