# Supplementary material for: Adverse pro-tumorigenic effects of IDO1 catalytic inhibitors mediated by the non-enzymatic function of IDO1 in tumor cells
Source: Front Immunol. 2025 Nov 4;16:1680896. doi: 10.3389/fimmu.2025.1680896 (PMC12623407; doi:10.3389/fimmu.2025.1680896)

Rossini *et al.* Raw figure 1B

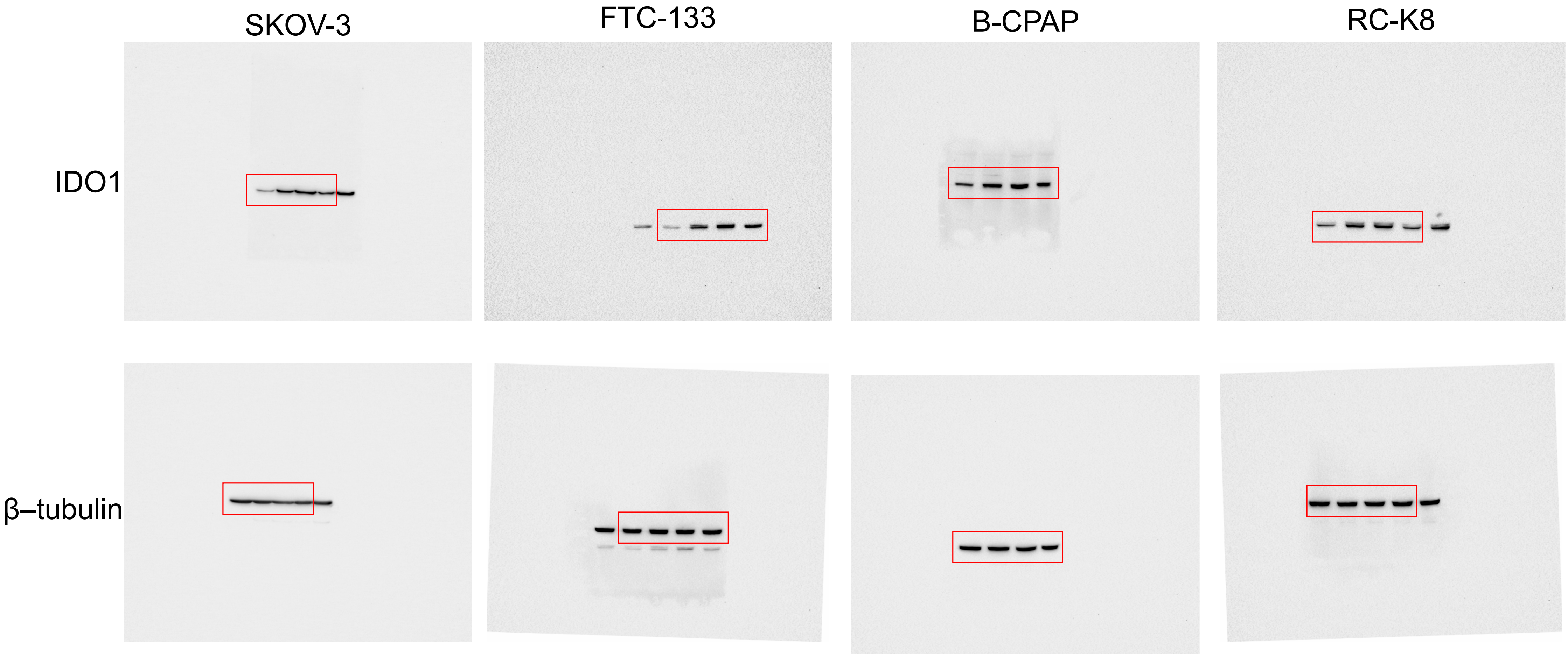

Rossini *et al.* Raw figure 2A

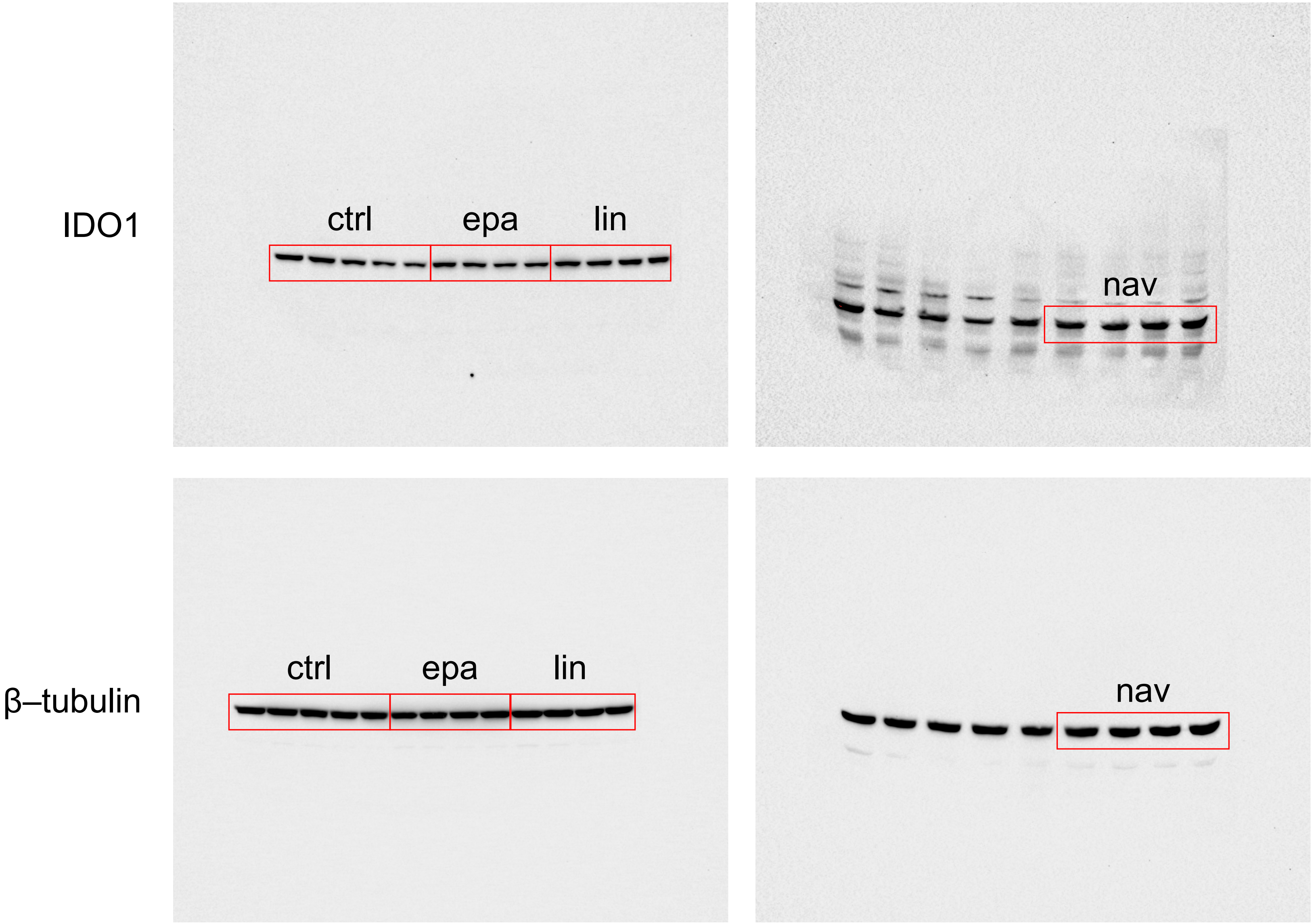

Rossini *et al.* Raw figure 2B

IDO1

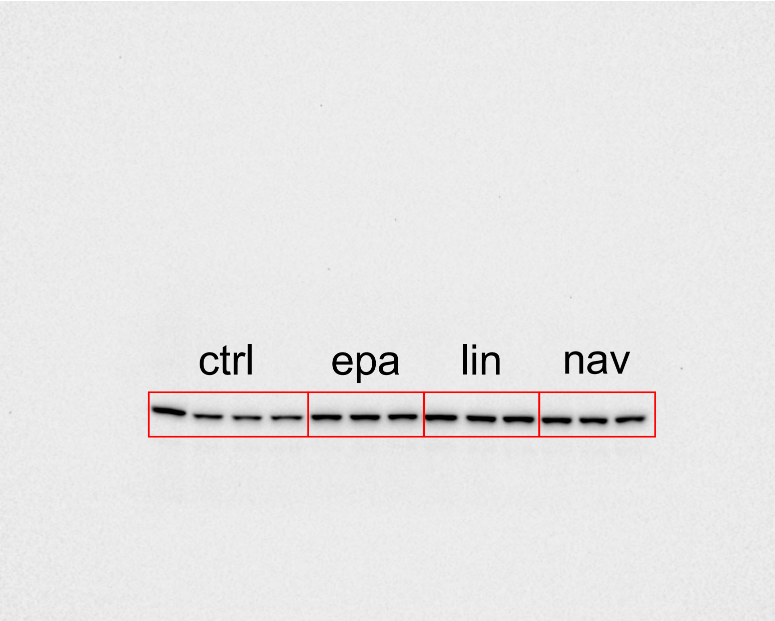

β–tubulin

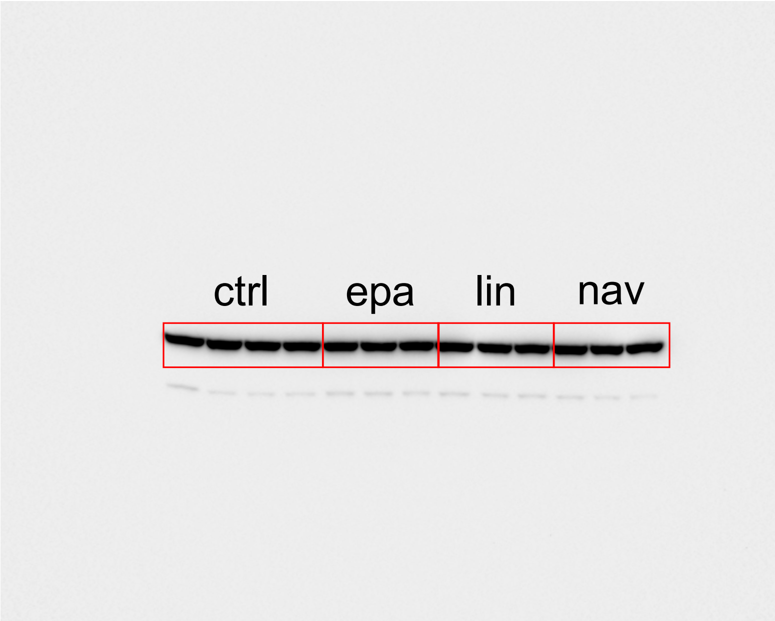

Rossini *et al.* Raw figure 3A

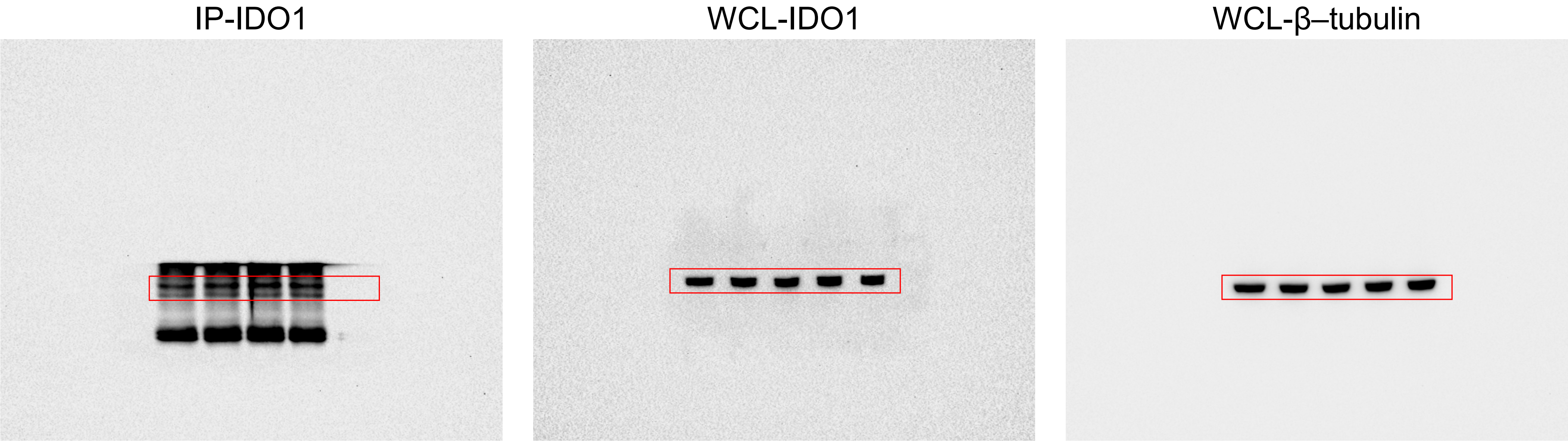

Rossini *et al.* Raw figure 3B

p-Src

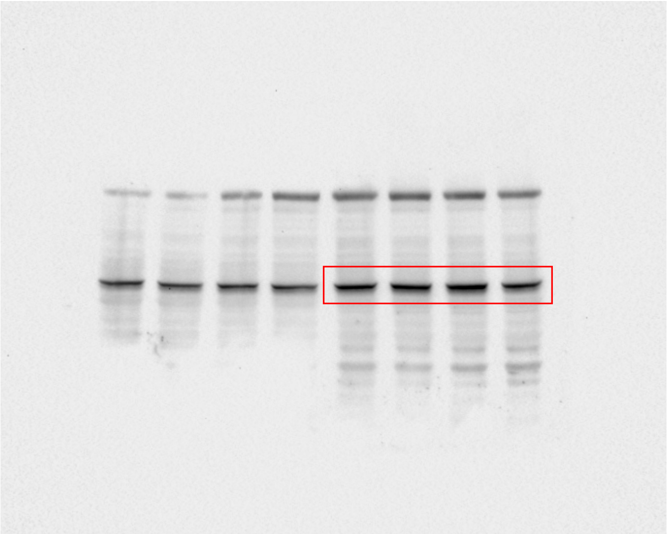

Src

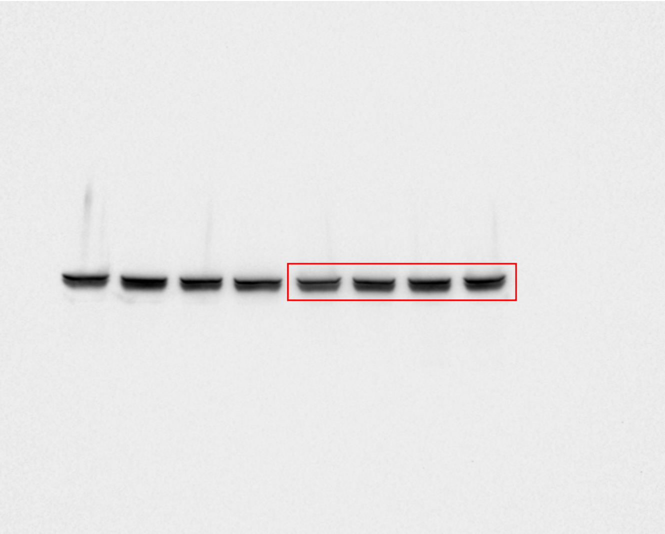

Actin

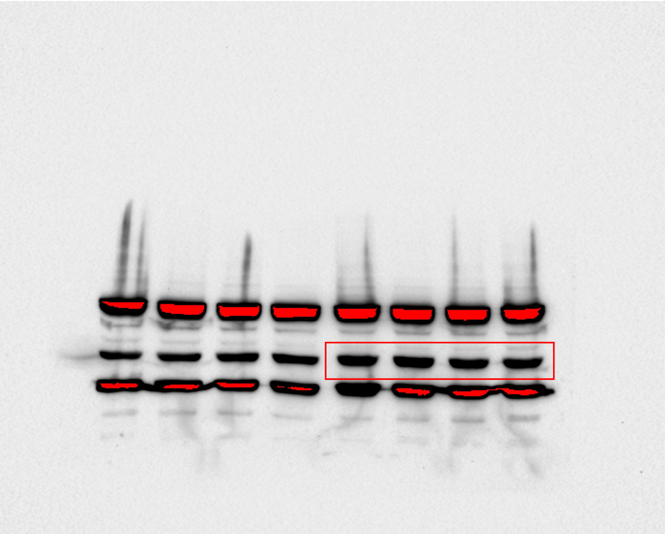

Rossini *et al.* Raw figure 3C

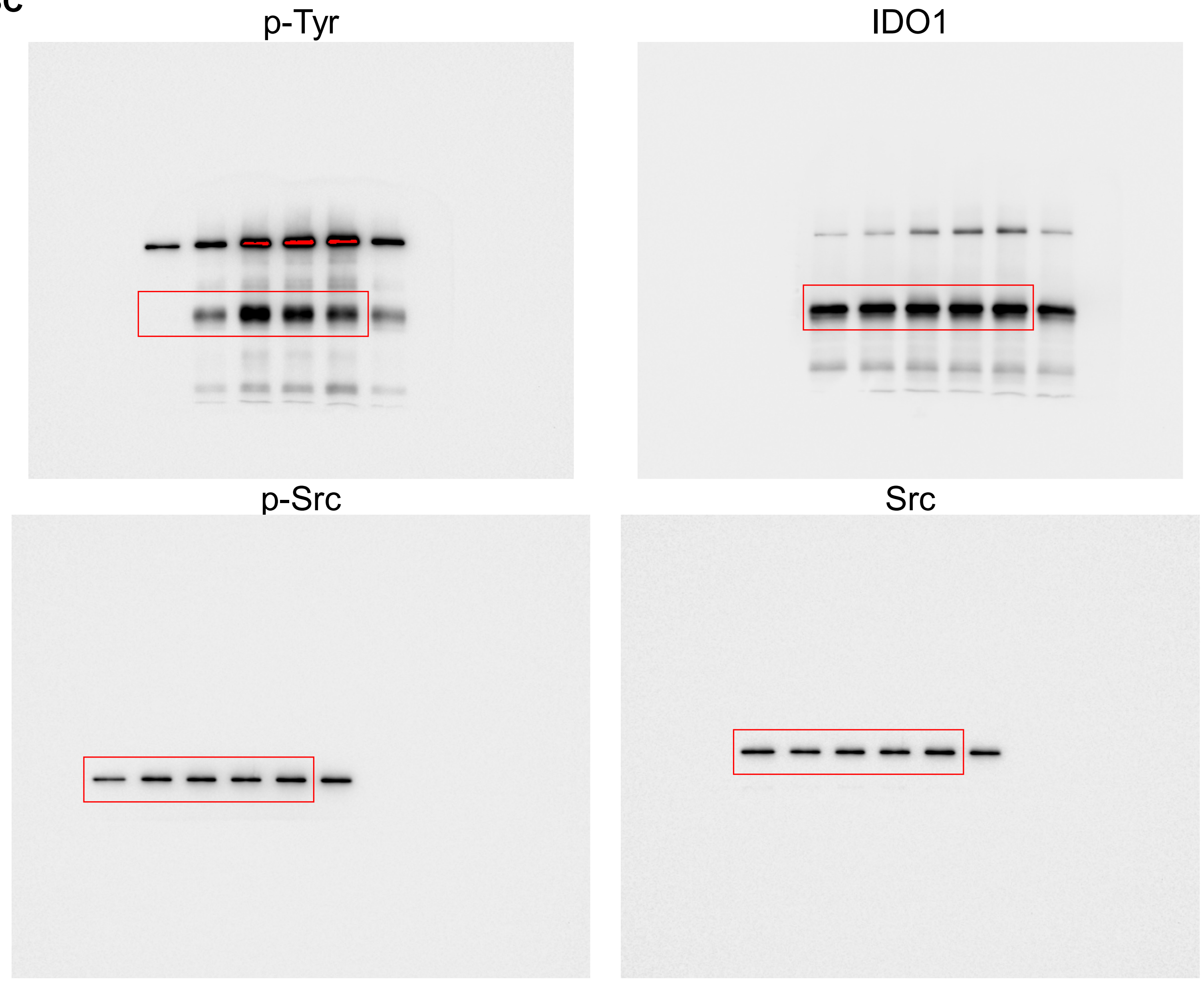

Rossini *et al.* Raw figure 5A

IDO1 β-tubulin

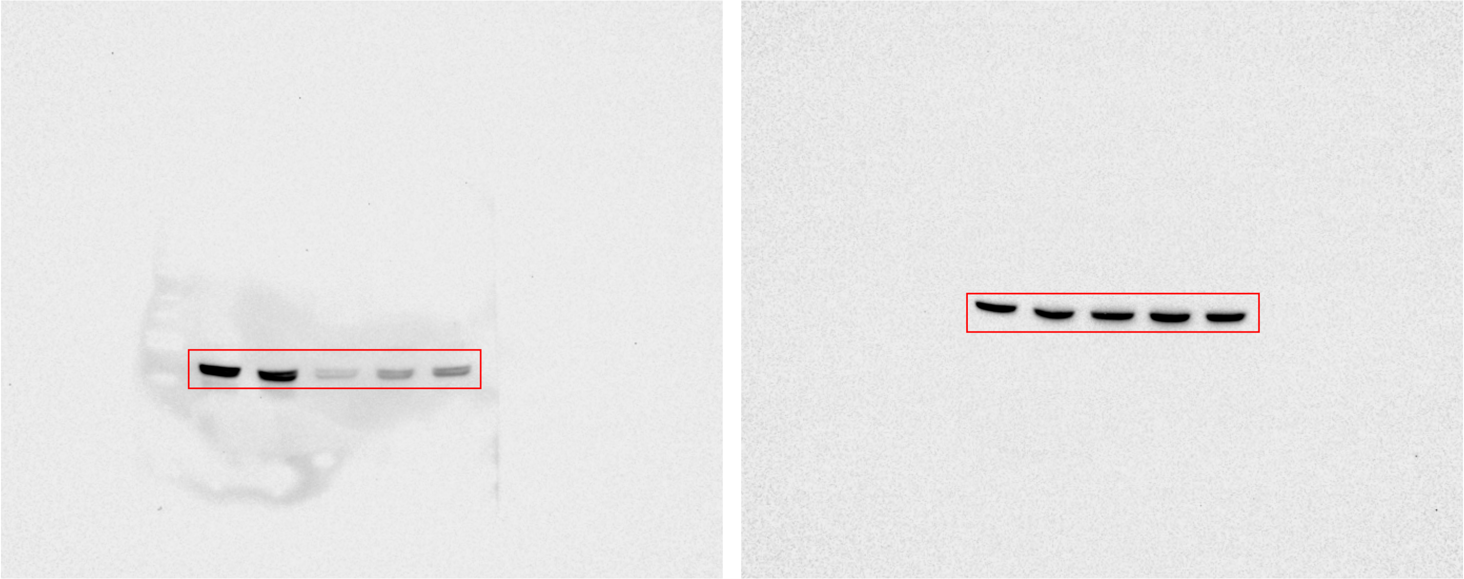

Rossini *et al.* Raw figure 6A

IDO1

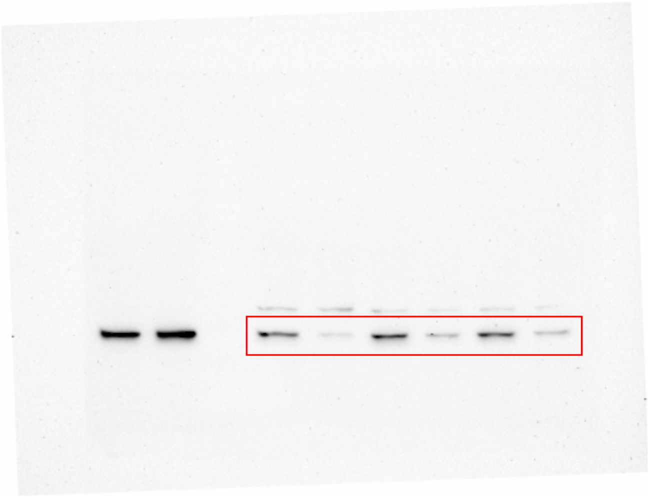

β-tubulin

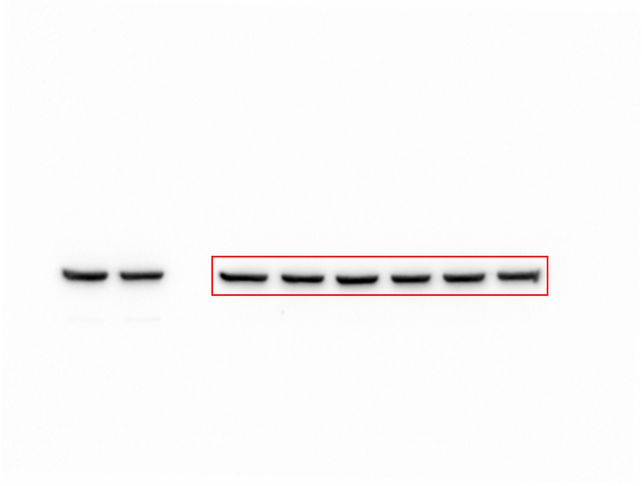

Rossini *et al.* Raw figure Supplementary 2A

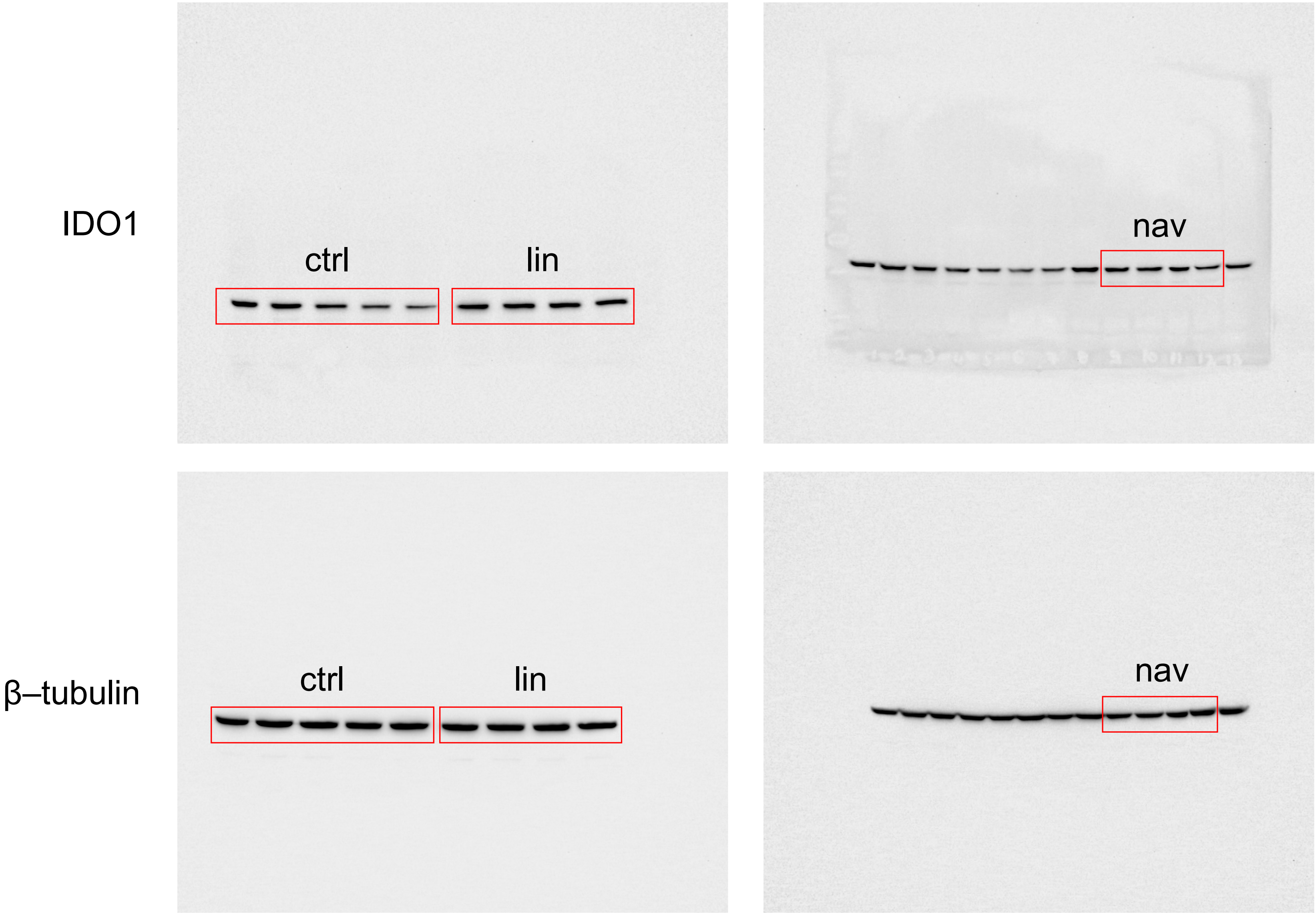

Rossini *et al.* Raw figure Supplementary 3A

IP-IDO1

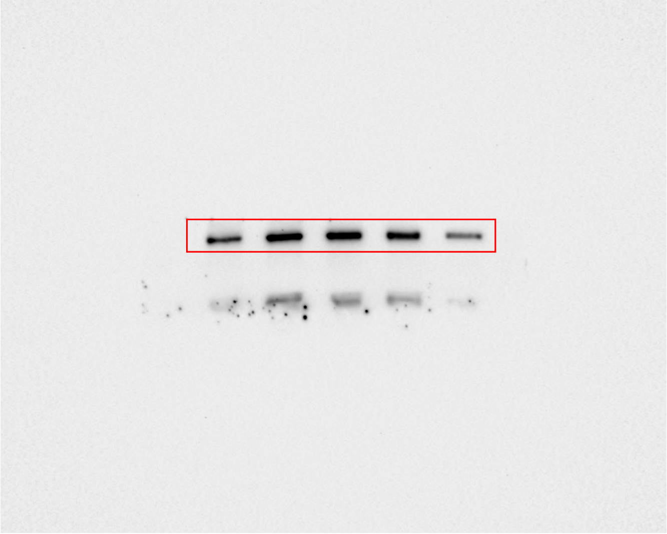

IP-Src

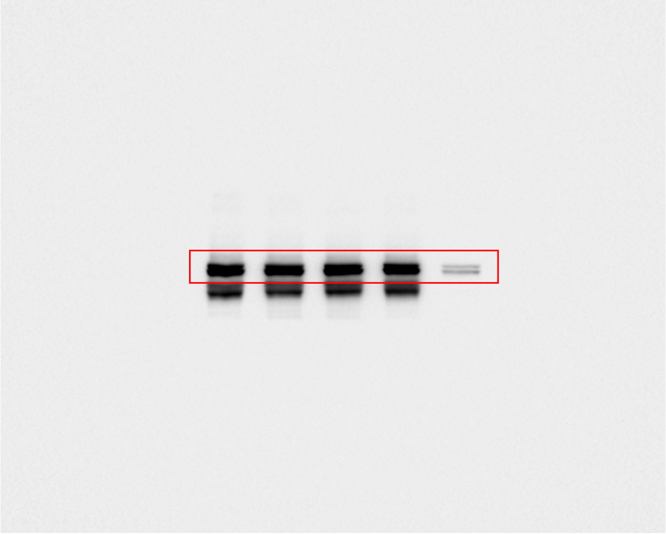

WCL-IDO1

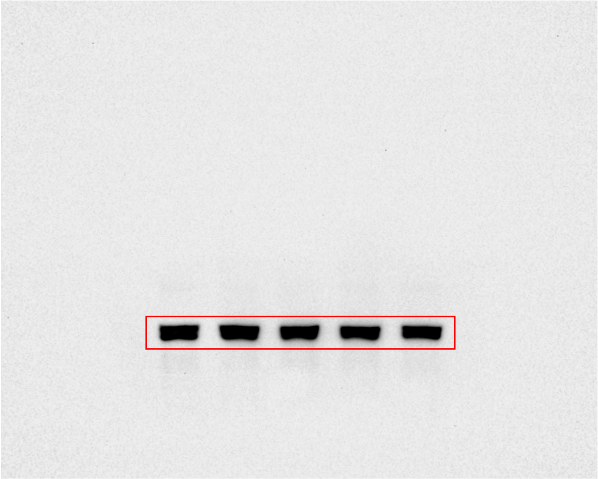

WCL-Src

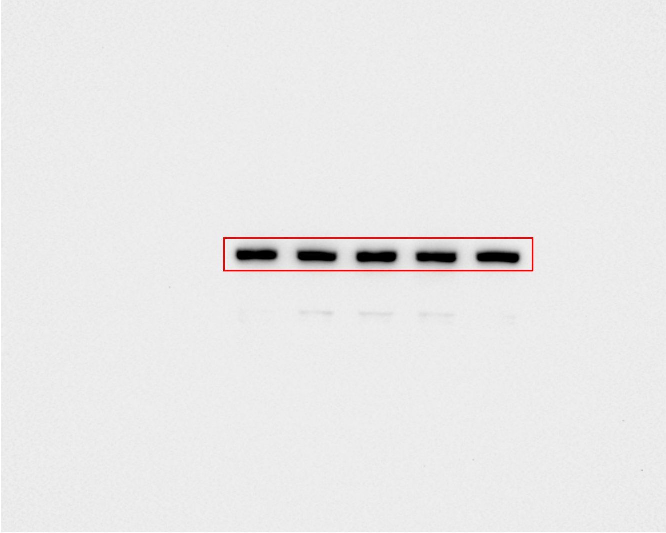

WCL-Gapdh

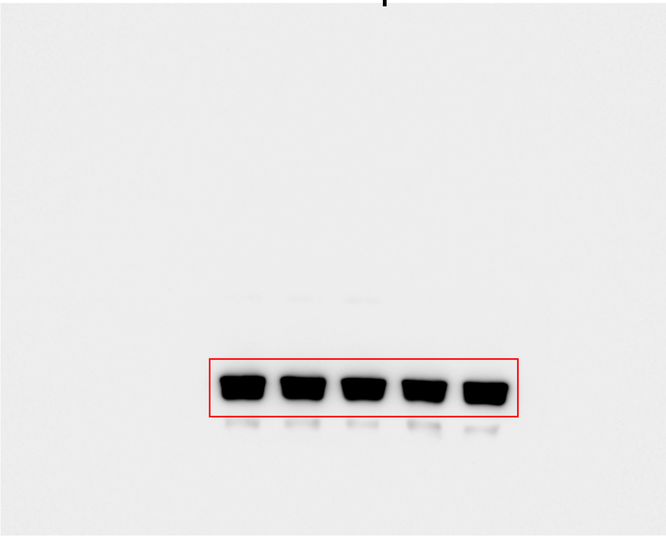

Rossini *et al.* Raw figure Supplementary 3B

IP-IDO1

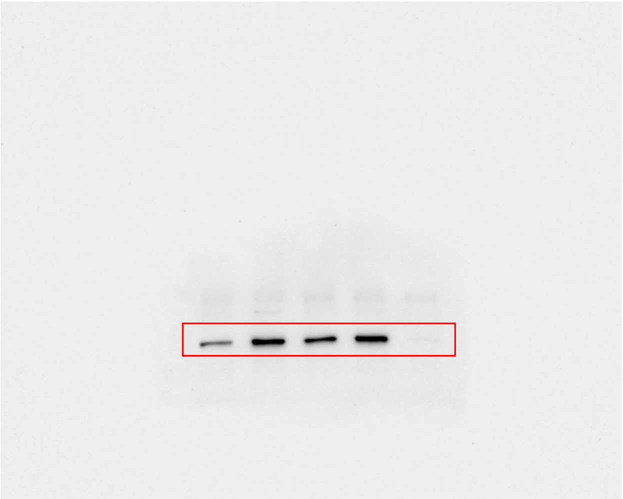

IP-SHP-2

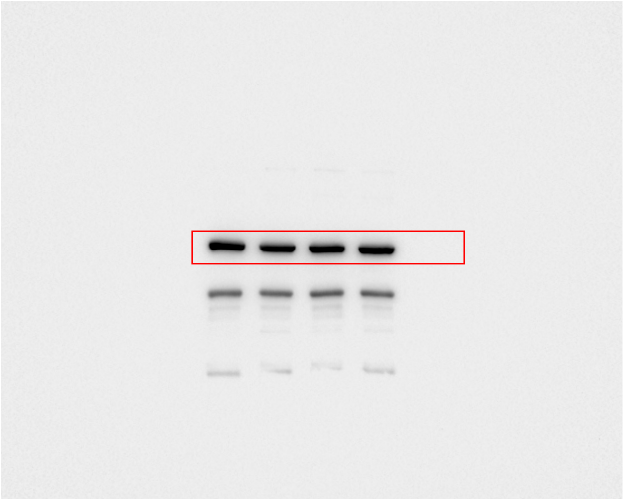

WCL-IDO1

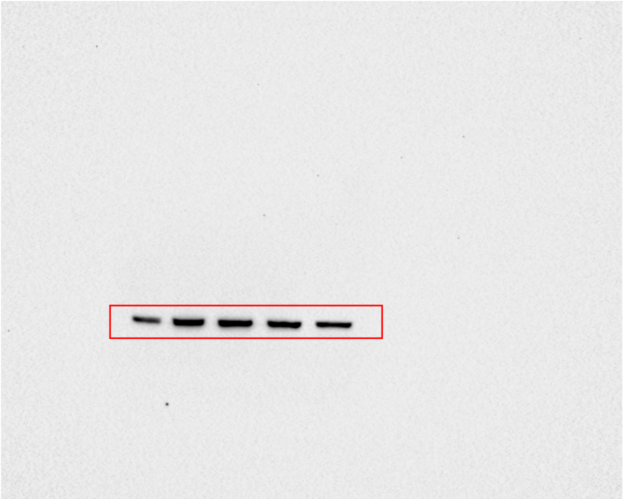

WCL-SHP-2

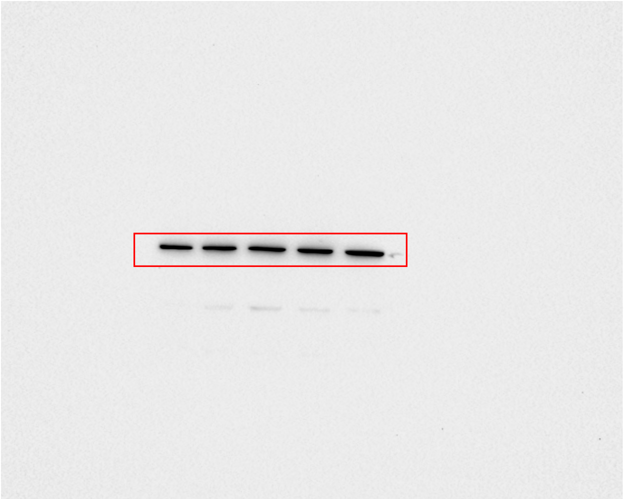

WCL-β-tubulin

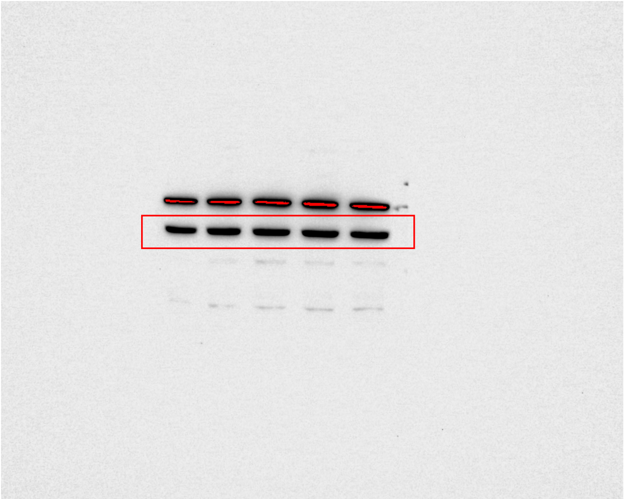

Supplement: Supplementary file 2 [file DataSheet2.pdf]
